# Anthocyanin-Rich Berry Extracts Affect SN-38-Induced Response: A Comparison of Non-Tumorigenic HCEC-1CT and HCT116 Colon Carcinoma Cells

**DOI:** 10.3390/antiox13070846

**Published:** 2024-07-15

**Authors:** Cornelia Schmutz, Crepelle Plaza, Franziska Steiger, Natascha Stoirer, Judith Gufler, Gudrun Pahlke, Frank Will, Walter Berger, Doris Marko

**Affiliations:** 1 Department of Food Chemistry and Toxicology, Faculty of Chemistry, University of Vienna, Währingerstraße 38-40, 1090 Vienna, Austria; cornelia.schmutz@univie.ac.at (C.S.); gudrun.pahlke@univie.ac.at (G.P.); 2 Doctoral School in Chemistry, University of Vienna, Währingerstraße 42, 1090 Vienna, Austria; 3 Department of Beverage Research, Hochschule Geisenheim University, P.O. Box 1154, 65366 Geisenheim, Germany; frank.will@hs-gm.de; 4 Center for Cancer Research and Comprehensive Cancer Center, Medical University of Vienna, Borschkegasse 8a, 1090 Vienna, Austria; walter.berger@meduniwien.ac.at

**Keywords:** DNA damage, irinotecan, NF-κB pathway, polyphenols, topoisomerase poison

## Abstract

Chemotherapy with irinotecan (CPT-11), the pro-drug of the highly cytotoxic SN-38, is among the standard-of-care treatments for colorectal cancer. To counteract undesired toxic side effects on healthy tissue such as the intestinal epithelium, the use of preparations rich in polyphenols with anti-oxidative and anti-inflammatory properties such as anthocyanins has been proposed. In the present study, the question of whether non-tumorigenic human epithelium cells (HCEC-1CT) can be protected against the cytotoxic impact of SN-38 by anthocyanin-rich polyphenol extracts without compromising the desired therapeutic effect against tumor cells (HCT-116) was addressed. Hence, single and combinatory effects of anthocyanin-rich polyphenol extracts of elderberry (EB), bilberry (Bil), blackberry (BB) and black currant (BC) with the chemotherapeutic drug SN-38 were investigated. Out of the extracts, BB showed the most potent concentration-dependent cytotoxicity alone and in combination with SN-38, with even stronger effects in non-tumorigenic HCEC-1CT cells. In cytotoxic concentrations, BB decreased the level of DNA/topoisomerase I covalent complexes in HCEC-1CT cells below base level but without concomitant reduction in SN-38-induced DNA strand breaks. The herein reported data argue towards an interference of anthocyanins with successful treatment of cancer cells and a lack of protective properties in healthy cells.

## 1. Introduction

**Figure 1:**
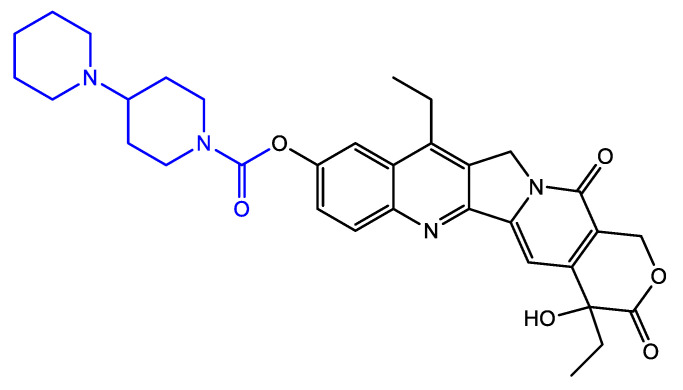
Chemical structure of irinotecan (whole structure, CPT-11, 7-ethyl-10[4-(1-piperidino)-1-piperidino] carbonyloxy-camptothecin) and its toxic metabolite SN-38 (black structure, 7-ethyl-10-hydroxy-camptothecin). Carboxylesterases are responsible for the cleavage of the carboxylic ester at position 10 (blue structure), where a hydroxy group remains.

Nowadays, colorectal cancer (CRC) is the third most frequent malignancy worldwide causing cancer-related deaths (Siegel, 2023). Next to surgery and radiotherapy, chemotherapeutic treatments with combinations of 5-fluoruracil (5-FU) and leucovorin, 5-FU and irinotecan (CPT-11) or CPT-11 alone are often used [1]. CPT-11 is the pro-drug of the highly cytotoxic metabolite SN-38, obtained through cleavage by carboxylesterases, and acts as a potent topoisomerase (topo) I poison (Figure 1). As such, it stabilizes the covalent DNA/topo I complex and leads to apoptosis, since the replication fork collides with the covalently bound complex [2,3]. As a camptothecin-derived chemotherapeutic, CPT-11 shares its pharmacologically unique properties. For instance, topo I is the only affirmed antineoplastic target of CPT-11 so far [4]. However, over the past few years, other proteins such as the E3 ligase MDM2 (ligase of tumor suppressor protein p53) and the multifunctional RNA- and DNA-binding protein FUBP1 (far-upstream element-binding protein 1) have been identified as potential targets of camptothecin-derived drugs [5]. Still, their potent and specific topo I inhibition together with their rapidly occurring penetration of vertebrate cells are seen as responsible for their anti-cancer activities. However, relatively high drug concentrations must be used to reach desired therapeutic effects, showing that camptothecin-derived drugs are well established for their selectivity rather than their potency [4]. As chemotherapeutics not only attack tumor cells but also healthy tissue, adverse effects of treatment with CPT-11 include neutropenia, mucositis and dose-limiting late-onset or bloody diarrhea [2,3]. These adverse effects are often contingent on the detoxification efficiency of individuals. The main pathway of SN-38 inactivation is through glucuronidation via uridine diphosphate-glucuronosyltransferases (UGT) 1A1 and 1A9, resulting in inactive SN-38 glucuronide, which is excreted with urine or feces. However, polymorphisms, resulting in very low or even non-existent UGT1A1 activity, were associated with higher risk of severe late-onsite diarrhea and leucopenia after CPT-11 treatment [6]. Furthermore, β-glucuronidase activity of the human microbiome located in the colon is responsible for reactivation of detoxified SN-38 glucuronide to SN-38, leading to increased local toxicity in the GIT and SN-38-related adverse effects [5]. Therefore, cancer patients often change their dietary habits or use dietary supplements additionally to their standard-of-care treatment hoping to ease undesired adverse effects [7]. Despite the prevalent intake of supplements by patients, only sparse information is available concerning positive or adverse effects of combinations of chemotherapeutic drugs and supplements. The use of supplements or a change in dietary habits might lead to higher uptake of bioactive compounds such as polyphenols.

As ubiquitously occurring secondary plant metabolites, polyphenols are widely discussed in the literature for potential anti-oxidative, anti-inflammatory, anti-cancer, anti-bacterial, anti-viral and even anti-Alzheimer’s properties [8]. Polyphenols can be divided into several structurally diverse subclasses. Anthocyanidins—belonging to the subclass of flavonoids—are the aglycones of anthocyanins, colored plant pigments responsible for the bright red to purple appearance of numerous vegetables and fruits. Systemic bioavailability of anthocyanins is reported to be low since less than 1.8% are absorbed in the gastrointestinal tract (GIT). On that account, local interactions of the food-associated compounds with the GIT are of particular interest [9]. Since anthocyanins are reported to interfere with processes involved in cancer initiation, promotion and progression, recent studies have focused on evaluation of their anti-proliferative effect on CRC [10,11,12,13]. Furthermore, anthocyanin-rich fruit extracts as well as single anthocyanins have been identified as catalytic inhibitors of topoisomerase I and II, thereby pointing out their potential to interfere with adverse effects of topoisomerase poisons such as CPT-11. So far, literature has focused on the interaction of these two substance classes in cancer cells while largely neglecting their effects on healthy tissue [14,15].

This study, therefore, aims to shed light on potential mechanisms involved in protective or adverse effects of anthocyanin-rich berry extracts from blackberry (BB, *Rubus fructicosus*), bilberry (Bil, *Vaccinium myrtillus*), black currant (BC, *Ribes nigrum*), and elderberry (EB, *Sambucus nigra*) on HCT116 colon carcinoma cells in comparison to non-tumorigenic human colonic epithelial HCEC-1CT cells in the context of SN-38-induced cytotoxicity, genotoxicity, oxidative stress and inflammation. Furthermore, the effects of single anthocyanins (Figure S1) that were found to be the most abundant in the respective extracts were investigated in the same experimental setups to determine if and to what extent they contributed to the extracts’ effects. In more detail, single anthocyanins were incubated in concentrations corresponding to the concentrations in the respective extract to facilitate drawing direct conclusions of observed effects.

## 2. Materials and Methods

### 2.1. Materials

SN-38 was purchased from Tocris Bioscience (Bristol, GBR). Anthocyanins cyanidin-3*O*-glucoside (Cy3glc), delphinidin-3*O*-glucoside (Del3glc), cyanidin-3*O*-rutinoside (Cy3rut) and cyanidin-3*O*-sambubioside (Cy3sam) were obtained from Extrasynthese (FRA, Rhône, France). Catalase from bovine liver, water-soluble hydrocortisone and 2′,7′-dichlorodihydrofluorescein diacetate (H_2_DCFDA) were purchased from Sigma-Aldrich (Saint Louis, MO, USA). Fetal calf serum (FCS), 1 M HEPES buffer solution, gentamicin solution, 10× Medium 199 and 100× insulin-transferrin-selenium-G (ITS) were obtained from Thermo Fisher Scientific (Waltham, MA, USA). Recombinant human EGF and cosmic calf^®^ serum were purchased from Corning^®^ Inc. (Corning, NY, USA) and GE Healthcare Life Sciences (Piscataway, NJ, USA), respectively. Zeocin^®^ and Normocin^TM^ were obtained from InvivoGen (San Diego, CA, USA). Detailed extract preparation and composition are provided in our previous publication [15]. Briefly, organic bilberry juice (Bayernwald Früchteverwertung KG, Hengersberg, Germany) and resulting juices from frozen, loose-rolling blackberries, elderberries and black currants (Trautner Fruit Trade, Berlin, Germany) were heated to 50 °C enzymation temperature and, subsequently, depectinized. Bilberry juice was loaded on SP70 resin (Resindion, Binasco, Italy), and blackberry, elderberry and black currant juice were loaded on ADS5000 resin (Chemra, Trier, Germany). Unwanted water-soluble compounds (e.g., fruit acids, minerals and sugars) were removed with distilled water. Juice polyphenols containing anthocyanins were then desorbed with ethanol. Extracts were concentrated and, subsequently, freeze dried. Anthocyanin-rich polyphenol extracts were stored under cool, dark and dry conditions to enhance stability. Anthocyanin concentrations of anthocyanin-rich polyphenol extracts were analyzed using HPLC (Accela, Thermo Fisher, Karlsruhe, Germany) with an external Cy3glc calibration. Anthocyanins were additionally assigned safely according to their mass spectra (Thermo Finnigan LXQ, Thermo Fisher, Karlsruhe, Germany). The most abundant anthocyanins were Cy3glc (88.5% of all anthocyanins in BB), Del3lgc (13.5% in Bil), Cy3rut (39.4% in BC) and Cy3sam (72.9% in EB), which were then used in concentrations corresponding to their amount in the extracts (Table 1) to investigate the potential effects of the single compounds.

### 2.2. Cell Culture

The non-tumorigenic human colonic epithelial cell line 1CT (HCEC-1CT) was kindly provided by Prof. Jerry W. Shay (UT Southwestern Medical Center, Dallas, TX, USA). HCEC-1CT cells were grown in Dulbecco’s Modified Eagle Medium (DMEM, high glucose, Thermo Fisher Scientific, Vienna, AUT) supplemented with 2% HyClone^TM^ Cosmic Calf^TM^ serum, 2% 10× Medium 199, 20 mM HEPES buffer, 50 μg/mL gentamicin, 1 μg/mL hydrocortisone, 10 μL/mL ITS supplement and 20 ng/mL recombinant human EGF. HCT116 cells were obtained from ATCC (Manassas, VA, USA) and cultivated in DMEM Glutamax^TM^ (Thermo Fisher Scientific) supplemented with 10% FCS and 1% penicillin/streptomycin (P/S, Thermo Fisher Scientific). THP1-Lucia^TM^ NF-κB reporter monocytes (InvivoGen, San Diego, CA, USA) were kept in Rosewell Park Memorial Institute (RPMI) 1640 medium supplemented with 10% FCS, 1% P/S, 25 mM HEPES and 100 µg/mL Normocin^TM^. Every second passaging, 100 µg/mL Zeocin^®^ was added to eliminate non-functional THP1-Lucia^TM^ reporter cells from the culture. The cells were kept in a humidified atmosphere with 5% CO_2_ at 37 °C, subcultured twice per week and used for up to 25 cell passages.

### 2.3. Treatment and Dosage Information

Anthocyanins/extracts and SN-38 were weighed, dissolved in DMSO, aliquoted and stored at −80 °C or −20 °C, respectively, to avoid repeated freeze–thaw cycles. DMSO was chosen as an aprotic solvent to further stabilize anthocyanins. The highest stocks of extracts prepared had a concentration of 40 mg/mL. Investigated concentrations of anthocyanin-rich extracts (6.25–200 µg/mL; 1:200 dilution of stock) and their respective single anthocyanins are reachable with a healthy diet. Single anthocyanins were incubated in concentrations corresponding to the respective extract (Table 1). For incubations, a maximum DMSO concentration of 0.6% was used. In combinatory experiments, anthocyanins and extracts were pre-incubated for 30 min to facilitate the uptake by cells before exposing them to the more lipophilic SN-38. SN-38 concentrations were adjusted for each assay to reach a significant effect in the respective incubation time. Hence, much lower concentrations of SN-38 were needed to reach a significant effect in cytotoxicity experiments (72 h incubation) compared to the comet assay (1 h incubation). All experiments were conducted with the addition of catalase (100 U/mL) to avoid accumulation of H_2_O_2_.

### 2.4. Coupled WST-1 and SRB Assay

Cytotoxicity measurements were carried out according to our published protocol [15]. Briefly, 2000 (HCEC-1CT) or 4000 (HCT116) cells were seeded per well in a 96-well plate and grown for 24 h. The cells were then incubated for 72 h with extracts (6.25–200 µg/mL) or anthocyanins (corresponding concentrations to berry extracts shown in Table 1) with or without SN-38. Afterwards, water-soluble tetrazolium (WST-1, abcam, Forchheim, Germany) solution was added and incubated for 45 min. Absorption of the formed formazan dye by metabolically active cells was measured with a microplate reader at 450 nm (reference wavelength of 650 nm). Subsequently, the cells were fixed with trichloroacetic acid (5%, *w*/*v*) at 4 °C, stained with sulforhodamine B (SRB, Alfa Aefsar, Tewksbury, MA, USA) solution for 1 h and the absorbance at 570 nm was measured after dissolving the dye in 10 mM TRIS Base buffer (Carl Roth, Karlsruhe, Germany).

### 2.5. Interaction Analysis

The mathematical model of independent joint action (IJA) is a widely used model to facilitate the interpretation of compound interactions. According to Formula (1), where *f_ab_* stands for the expected combined effect and *f_a_*, *f_b_* are the measured effects of the single substances, the expected additive effect of substance interactions was calculated:

(1) $$f_{ab}=f_{a}+f_{b}-f_{a}*f_{b}$$

To determine if the measured combined effect is the result of synergistic, additive or antagonistic interactions, it is compared to the calculated combined effect (=1 − *f_ab_*) via a two-sample Student’s *t*-test [16,17].

### 2.6. In Vivo Complex of Enzyme (ICE) Assay

A total of 4 × 10^6^ (HCEC-1CT) or 3 × 10^6^ (HCT116) cells were seeded onto petri dishes with a diameter of 15 cm and grown for 72 h, followed by 30 min pre-incubation with single anthocyanins or extracts (100 µg/mL) and subsequent co-incubation with 1 µM SN-38 for 1 h. The ICE assay was conducted according to previously published protocol by Subramanian et al. [18]. Briefly, cells were washed with PBS, lysed (1% sodium lauroylsarcosylate) and garnered. The cell lysate was then pipetted on a cesium chloride density gradient (0.75–1.6 mg/mL in TE buffer, Carl Roth, Germany) and centrifuged for 22 h at 21 °C and 23,600 rpm (=100,000× *g*) to separate the heavier bound DNA/topo complexes from the free topoisomerase enzyme. After fractionation, the DNA content was measured with a Nano-Drop 2000 (Thermo Fisher Scientific), and fractions were blotted on a nitrocellulose membrane (4.5 µm, Amersham^TM^ Protran^TM^, Sigma-Aldrich). The membrane was then blocked with milk powder solution (Carl Roth, Germany) for 1.5 h, followed by overnight staining with an anti-topo I antibody (1:1000, rabbit, polyclonal, A12161, antibodies.com, Cambridge, GBR) at 4 °C. The secondary antibody was HRP-coupled (1:500, mouse anti-rabbit, sc-2357, Santa Cruz Biotechnology, Santa Cruz, CA, USA) and reacted with the ECL^TM^ Western blotting detection solution (Amersham^TM^, Sigma-Aldrich) after 2 h of incubation at room temperature. Chemoluminescent signals were detected with an LAS-4000 imager (Fujifilm Life Science, Duesseldorf, Germany) and evaluated with MultiGauge software (version 1.0, Fujifilm Life Science, Germany).

### 2.7. Comet Assay

Single-cell gel electrophoresis was conducted according to previously published protocols [15,19]. Shortly, 250,000 cells were seeded in a 35 mm petri dish and grown for 24 h. After pre-incubation with extracts (0.1–200 µg/mL) or anthocyanins for 30 min, SN-38 at a concentration of 5 µM was co-incubated for one hour. Cells of the positive control were irradiated with UV-B light (41 J, 1 min). After harvesting of the cells, the cell count and viability were determined via trypan blue exclusion. A total of 30,000 cells of each sample were embedded onto two microscopy slides in 0.8% low-melting agarose in duplicates. Cell lysis was performed overnight at 4 °C (pH 10, 1% lauroylsarcosylate, 10% DMSO, 1% Triton-X 100; Carl Roth, Germany). Half of the slides were treated with formamidopyrimidine-DNA glycosylase (FPG; New England Biolabs, Frankfurt, Germany) for 30 min at 37 °C. Electrophoresis was carried out after 20 min of equilibration in electrophoresis buffer (pH 13, NaOH, EDTA). Electrophoretic parameters were set as follows: 0 °C, 300 ± 3 mA and 25 V (0.028 V/cm^2^) for 20 min. After neutralization, the slides were stained with ethidium bromide solution (0.02 mg/mL, Carl Roth, Germany) and analyzed with a Zeiss Axioskop (λ*_ex_* 546 ± 1 nm, λ*_em_* ≥ 590 nm). Software-supported analysis was performed with Comet Assay IV (version 4.2.1; Perceptive Instruments, Potsdam, Germany). Per condition, 100 nuclei were scored and used for evaluation.

### 2.8. Dichlorofluorescein (DCF) Assay

Cells were seeded at a density of 35,000 (HCT116) or 20,000 (HCEC-1CT) and grown for 24 h. The DCF assay was carried out according to the method by Wang and Joseph [20]. Briefly, cells were incubated for 15 min at 37 °C with 50 µM H_2_DCFDA solution. This solution was then removed, and the cells were washed twice with PBS. In the second washing step, the background fluorescence of each well was measured (λ_*ex*_ 485 nm, λ_*em*_ 528 nm). Increasing concentrations of the test solutions (25–200 µg/mL berry extracts) in phenol red-free medium were then pipetted onto the cells, and the fluorescence signal was measured immediately with a plate reader (λ_*ex*_ 485 nm, λ_*em*_ 528 nm) at six time points (0, 15, 30, 45, 60 and 90 min). For induction of oxidative stress, 1 mM H_2_O_2_ was used as a positive control. Additionally, the protective DCF (pDCF) assay previously described by Pahlke et al. was applied [21]. HCT116 or HCEC-1CT cells were seeded, grown for 24 h and then incubated with increasing concentrations of the test substances for 24 h. After incubation with H_2_DCFDA solution and measurement of the background signal as described for the DCF assay, cells were challenged with 1 mM H_2_O_2_ solution, and the fluorescence signal was measured immediately with a plate reader (λ_*ex*_ 485 nm, λ_*em*_ 528 nm) at six time points (0, 15, 30, 45, 60 and 90 min). Cells not challenged with H_2_O_2_ served as a control for sufficient signal induction by H_2_O_2_.

### 2.9. NF-κB Reporter Gene Assay

The assay protocol was adapted from Woelflingseder et al. [22]. Increasing concentrations of the test substances (6.25–200 µg/mL of berry extracts and corresponding concentrations of single anthocyanins shown in Table 1) were pipetted into a 96-well plate to be further diluted 1:2 by the addition of THP1-Lucia^TM^ cells. For this assay, the use of catalase was omitted due to unforeseen interference with the measurement. THP1 cells were counted, and an adequate volume of cell suspension containing 100,000 cells for each well was transferred into a tube to be centrifuged at 140 rcf for 2 min at RT. The supernatant was removed and the cell pellet resuspended to yield 100,000 cells per 100 µL. With a multichannel pipette, 100 µL of this suspension was added to each well of the plate containing the test substances at different concentrations. As a positive control for anti-inflammatory activity, 1 µM dexamethasone (Dex, Sigma-Aldrich, USA) was used. The plate was shaken on a plate shaker at 500 rpm for 1 min and then incubated for 2 h. Thereafter, LPS (from *E. coli*, Sigma-Aldrich, USA) was added to each well, except the solvent control, reaching a concentration of 10 ng/mL. The plate was again shaken on the plate shaker for 1 min at 500 rpm and finally incubated for further 18 h to allow the release of luciferase protein. The plate was centrifuged at 140 rcf for 2 min, and 10 µL of the supernatant was transferred from each well to an opaque 96-well plate. Measurement of the luminescence signal obtained by addition of QUANTI-Luc^TM^ (InvivoGen, USA), hence the substrate of luciferase, was conducted according to the manufacturer’s protocol. Furthermore, cell viability was monitored in parallel to the NF-κB assay in the same plate. Therefore, after removal of 10 µL of supernatant, 20 µL of CellTiter-Blue^TM^ (CTB, Promega, Madison, WI, USA) reagent was added to each well and incubated for 2 h. As a positive control for reduced cell viability, 0.1% Triton-X 100 (TX) was used. The plate was shaken for 1 min at 500 rpm and then centrifuged at 140 rcf for 2 min. A volume of 100 µL of supernatant from each well was transferred to an opaque plate, and the fluorescence signal was measured with a plate reader (λ_*ex*_ 560 nm, λ_*em*_ 590 nm).

### 2.10. qRT-PCR

HCEC-1CT (60,000 cells/well, 12-well plates) and HCT116 (70,000 cells/well, 24-well plates) were seeded and grown for 48 h. Cells were pre-incubated with increasing concentrations of the berry extracts (1, 10 and 100 µg/mL) for 2 h and subsequently co-incubated for 3 h with 25 ng/mL human recombinant IL-1β protein (InvivoGen, USA) to mimic intestinal inflammation. Cell lysis and RNA extraction were carried out according to the manufacturer’s protocols with the Maxwell^®^ simplyRNA Cells extraction kit (Promega, USA) for HCEC-1CT and the RNeasy kit (Qiagen, San Diego, CA, USA) for HCT116 cells. RNA concentration and purity were measured with the NanoDrop 2000. Thereafter, 1 µg of RNA was reversely transcribed using the QuantiTect Reverse Transcription Kit (Qiagen) according to the manufacturer’s instructions. Exponential amplification of gene-specific cDNA (initial input 20 ng) was carried out using the qRT-PCR technique on a StepOne Plus PCR System (Applied Biosystems, Thermo Fisher Scientific). MiScript SYBR Green PCR Kit (Qiagen) and the following mRNA specific QuantiTect^®^ primer assays (Qiagen) were used: β-actin (Hs_ACTB_1_SG, QT00095431), glyceraldehyde 3-phosphate dehydrogenase (GAPDH, HS_GAPDH_1_SG, QT00079247), cyclooxygenase 2 (COX-2, Hs_PTGS2_1_SG, QT00040586), IL-1*β* (Hs_IL1B_1_SG, QT00021385), IL-6 (Hs_IL6_1_SG, QT00083720), IL-8 (Hs_CXCL8_1_SG, QT00000322) and TNF-*α* (Hs_TNF_1_SG, QT00029162). The amplification protocol started with an activation step of the polymerase at 95 °C for 15 min, followed by 40 cycles of denaturation at 94 °C for 15 s, annealing at 55 °C for 30 s and extension at 70 °C for 30 s, finalized by a melting curve analysis. Data evaluation was conducted using the 2^−ΔΔCT^ method described by Livak and Schmittgen [23,24].

### 2.11. Statistical Analysis

The presented data are the result of at least three independent biological replicates, each measured in technical duplicates (qPCR, comet assay) or triplicates (cytotoxicity, DCF assay and NF-κB reporter gene assay). Only ICE assay results represent biological replicates of single sample measurements, since sample size restriction did not allow for technical replicates. Results presented are the mean + SD of all measured replicates. After performing the Nalimov outlier test and verifying normal distribution with the Kolmogornov–Smirnov test, statistical significances were determined with Student’s one- and two-sample *t*-tests as well as one-way ANOVA with post hoc Bonferroni test. Values were considered as significantly different if *p* ≤ 0.05, ≤0.01, ≤0.001.

## 3. Results and Discussion

### 3.1. Influence on Cytotoxicity

**Figure 2:**
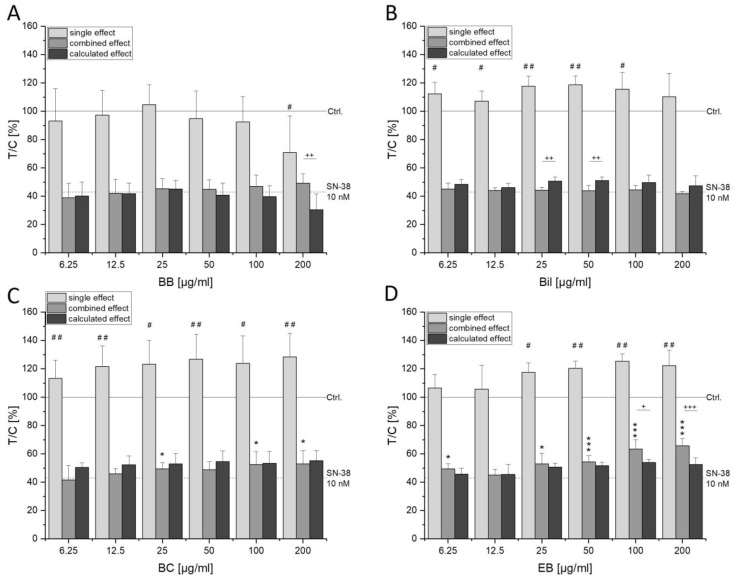
Cytotoxicity of blackberry (BB; **A**), bilberry (Bil, **B**), black currant (BC; **C**) and elderberry (EB; **D**) extracts in HCT116 cells after 72 h of exposure measured with water-soluble formazan (WST-1) assay. Results are depicted as mean + SD of 3-5 biologically independent replicates measured in technical triplicates expressed as test over control (T/C) relative to the solvent control (y = 100%, Ctrl., 0.6% DMSO). Cells were either incubated with extract alone (light gray bars) or together with 10 nM SN-38 (middle gray bars), and statistical differences were tested with one-sample (# *p* < 0.05, ## *p* < 0.01) or two-sample (* *p* < 0.05, *** *p* < 0.001) Student’s *t*-test, respectively. The dashed line indicates the mean value received after 72 h incubation with 10 nM SN-38 alone (43.0 ± 6.1%). Furthermore, the expected additive value (EV) of their combination (dark gray bars) was calculated using the model of independent joint action (IJA), and significant differences according to two-sample Student’s *t*-test were marked with + *p* < 0.05, ++ *p* < 0.01, +++ *p* < 0.001.

**Figure 3:**
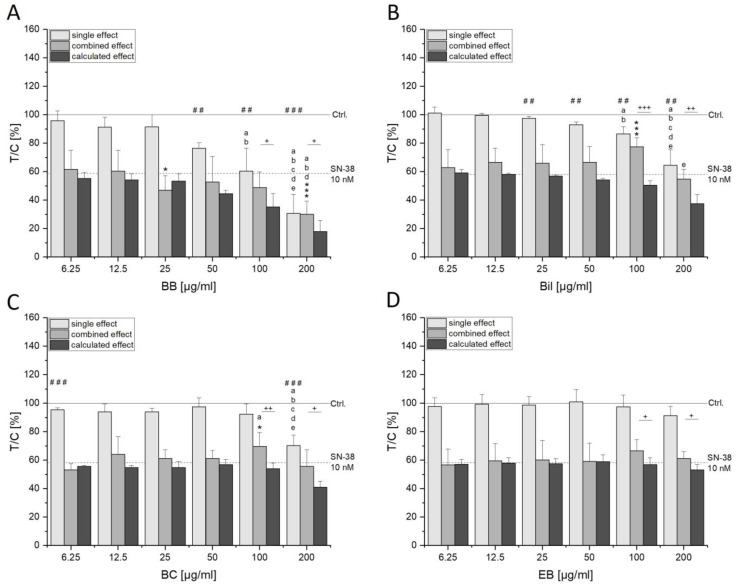
Cytotoxic properties of anthocyanin-rich extracts BB (**A**), Bil (**B**), BC (**C**) and EB (**D**) in HCEC-1CT cells measured with WST-1 assay. Cells were incubated for 72 h with increasing concentrations of extracts alone (light gray bars) or together with 10 nM SN-38 (middle gray bars). The dashed line indicates the mean value received after 72 h incubation with 10 nM SN-38 (58.3 ± 12.3%). Graphs show the mean + SD of 3–5 independent biological replicates measured in technical triplicates relative to the solvent control (y = 100%, Ctrl., 0.6% DMSO). Dark gray bars depict the EV calculated with IJA, and significant differences to the measured combined effects were calculated with two-sample Student’s *t*-test (+ *p* < 0.05, ++ *p* < 0.01, +++ *p* < 0.001). Statistical differences to the respective control were calculated with one-sample (## *p* < 0.01, ### *p* < 0.001) or two-sample (* *p* < 0.05, *** *p* < 0.001) Student’s *t*-tests. Differences among the concentrations were determined with one-way ANOVA via post hoc Bonferroni test (*p* < 0.05, a–e; e.g., the letter a represents significant difference to the lowest tested concentration of 6.25 µg/mL).

Cytotoxic properties of extracts alone and in combination with SN-38 were investigated after 72 h of incubation with the coupled WST-1 and SRB assay. In HCT116 cells, only 200 µg/mL BB extract showed a reducing effect on cell viability in the WST-1 assay, which was even more pronounced in the SRB assay (light gray bars, Figure 2 and Supplementary Figure S2). The remaining extracts seemed to even induce cell proliferation as observed through significantly higher signals compared to the control (Figure 2). However, a lower protein content could be found with these concentrations (Supplementary Figure S2) rather hinting towards metabolic stress leading to an increased signal in the WST-1 assay and not an enhancement of cell proliferation. Before assessing combinatory effects, the concentration-dependent decrease in cell viability mediated by increasing concentrations of SN-38 was investigated. In both cell lines tested, 10 nM SN-38 led to a significant decrease in cell viability as depicted by the dashed line in Figure 2, Figure 3, Figures S2 and S3. This concentration was, thus, chosen for investigation of combined effects with the single compounds. Mostly, combinations with anthocyanin-rich extracts did not significantly alter the toxic properties mediated by SN-38 in HCT116 cells. However, BC as well as EB extract showed significantly higher signals compared to the control (Figure 2, middle gray bars) without concomitant increase in cellular protein (Figure S2). The mathematical model of independent joint action (IJA) was applied to calculate combinatory effects. Based on the effect of the extracts and SN-38, an expected value was calculated for direct co-incubation. A comparison of the effects of co-incubation with the expected value (EV) allowed us to discriminate between additive, synergistic and antagonistic effects [16,17]. Over the whole concentration range, BC showed additive effects as IJA calculation of the EV (dark gray bars, Figure 2C) did not differ from the measured combined effects. Antagonistic interactions could be determined in both assays for 200 µg/mL BB and 100–200 µg/mL EB, while Bil showed opposing trends compared to the EV in the assays (Figure 2 and Figure S2).

HCEC-1CT cells seem even more sensitive towards the cytotoxic effects of the extracts. For instance, BB and Bil induced concentration-dependent cytotoxic effects from 25–200 µg/mL (Figure 3A,B). While EB showed only a slight, not significant tendency to decrease cell viability at 200 µg/mL, BC could reduce it to 70.3 ± 7.3%. In combination with SN-38, 200 µg/mL BB additionally decreased cell viability. Bil and BC at 100 µg/mL, conversely, seemed to protect against SN-38-induced cytotoxicity, conveying an antagonistic interaction (Figure 3). In the SRB assay, this antagonism could still be observed in tendencies. However, no significant protection from SN-38 was evident at the protein level (SRB assay), and high concentrations of the extracts rather tended to further reduce cell viability (Figure S3). Overall, the observed toxic effects mediated by the extracts could not be attributed to their main anthocyanins. Concentrations of the anthocyanins Cy3glc, Del3glc, Cy3rut and Cy3sam corresponding to their content in the respective extract did not reduce cell viability in HCT116 and HCEC-1CT cells nor influence SN-38-mediated cytotoxicity (Figure S4). On the contrary, HCEC-1CT cells showed increased cell protein content when exposed to the single anthocyanins (especially Cy3glc and Del3glc, Figure S4), leading to synergistic interaction potential since measured values were significantly lower than the EV. However, no significant effect compared to the SN-38 control could be observed (Figure S4B,D).

Not only does the non-tumorigenic HCEC-1CT cell line tend to be more sensitive towards exposure to the anthocyanin-rich extracts, but also a clear ranking of cytotoxic properties of the berry extracts could be determined: BB > Bil ≈ BC >> EB. In HCT116, conversely, BB could be identified as the most cytotoxic, with the other extracts not differing substantially in their potency, which was already observed in the murine colon cancer cell line CT26 [15]. Cytotoxic effects of anthocyanin-rich extracts on cancer-derived cell lines have been investigated in former studies. For instance, cytotoxic effects of a blueberry extract were observed after 72 h incubation starting from 12 and 25 mg/L gallic acid equivalents in CT26 and HCT116 cells, respectively [25]. Comparable to HCT116 cells, 100 µg/mL blackberry extract reduced cell viability to 60% in RAW 264.7 macrophages. However, this effect was already observed after 24 h of incubation, hinting towards an even higher cytotoxic potential of that extract [26]. In combination with the tyrosine kinase inhibitor erlotinib, a bilberry extract showed antagonistic interaction potential over the whole concentration range in A431 cells [27]. In the present study, comparable results were obtained with the Bil extract in the SRB assay, which showed antagonistic effects from 25–200 µg/mL in HCT116 cells (Figure S2). Additionally, by combination of SN-38 with the BB or EB extract, the EV could not be reached. However, since no significant difference to the SN-38-induced cytotoxic effect could be observed in the cancer cell line, the extracts do not seem to interfere with the effectiveness of the chemotherapeutic treatment in HCT116 cells. Of note, data on the effects of the respective polyphenols or the combination of them with chemotherapeutics in non-tumorigenic cells are far more limited. For instance, a blackberry extract and its major anthocyanin Cy3glc were tested in immortalized HaCat keratinocytes after 48 h of exposure. In alignment to our results, only the extract but not Cy3glc was found to be cytotoxic in the SRB assay [28]. However, the potency of the observed cytotoxic effects differs strongly. Only the highest concentration of the applied blackberry extract (100 µM) could diminish HaCat cell viability slightly by about 20% [28], whereas the BB extract used in the present study mediated potent concentration-dependent cytotoxic effects in HCEC-1CT cells starting from 25 µg/mL, reaching a maximum at 200 µg/mL with 10.7 ± 4.9% of cell viability in the SRB assay (Figure S3). Protective effects of anthocyanins are widely discussed in the literature [29,30,31]. However, in the present study, neither low, non-cytotoxic concentrations nor higher concentrations of the extracts or respective characteristic anthocyanins could convincingly protect non-tumorigenic HCEC-1CT cells from SN-38-mediated cytotoxicity. Rather, the opposite could be observed for the BB extract as well as the highest applied concentrations of Bil and BC.

### 3.2. Influence on the Formation of DNA/Topo I Intermediates

**Figure 4:**
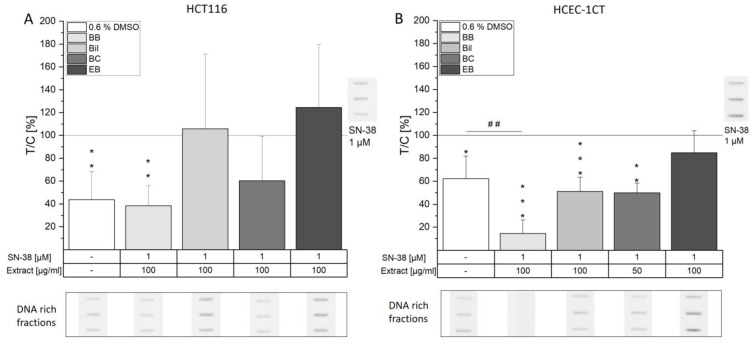
ICE assay detection of covalently bound DNA/topo I intermediates in HCT116 (**A**) and HCEC-1CT (**B**) cells. Cells were pre-incubated with extracts for 30 min, followed by co-incubation with 1 µM SN-38 for 1 h. A representative immunoblot from 3-5 biological replicates depicting the DNA-rich fractions used for evaluation is shown with equally modified contrast to improve visualization. Data shown are the means + SD evaluated as T/C of the SN-38 control in %. Significances compared to the SN-38 control were calculated with one-sample Student’s *t*-tests (* *p* < 0.05, ** *p* < 0.01, *** *p* < 0.001), and significant differences to the solvent control were evaluated with two-sample Student’s *t*-tests (## *p* < 0.01).

Topoisomerases (topo) are not only the target of several chemotherapeutic drugs such as doxorubicin or irinotecan, but also, anthocyanins have been reported to inhibit topo function. Doxorubicin and irinotecan are well known to act as so-called topo poisons, stabilizing covalent DNA/topo complexes, which are formed during the catalytic cycles of topos. In contrast, anthocyanins have been identified as potential catalytic topo inhibitors, suppressing the formation of the crucial covalent DNA/topo complex [14,32]. As a consequence, the presence of anthocyanins might decrease the level of the target structures of topo poisons, an effect with the potential to decrease the DNA-damaging properties of these chemotherapeutics. However, overbearing suppression of the catalytic activity of topos might also result in DNA damage due to failure in the regulation of DNA topology. Taking this as a starting point, the question of whether and to what extent the anthocyanin-rich extracts interfere with SN-38-mediated topo I poisoning in tumorigenic and non-tumorigenic cells was addressed. The cells were pre-incubated for 30 min with the extracts, subsequently co-incubated for another hour with 1 µM SN-38 and then deployed to the ICE assay procedure to determine the level of covalent DNA/topo I complexes. Concentrations of the extracts were chosen based on trypan blue exclusion tests after 1.5 h of incubation with a threshold of 80% cell viability. Of note, toxic effects of BC only allowed the testing of 50 µg/mL in HCEC-1CT cells, since at 100 µg/mL, a cell viability of about 52% was observed. SN-38 (1 µM) significantly induced the level of DNA/topo I intermediates in both cell lines (Figure 4). The signal obtained by the solvent control can be interpreted as basal cell level of formed covalent complex necessary for normal cell function. In HCT116 cells, neither Bil nor EB extract affected SN-38-induced DNA/topo I intermediate levels. With the BC extract, a non-significant tendency to reduce the signal could be observed. Solely the BB extract potently prevented DNA/topo I intermediate stabilization, as the signal remained at solvent control level and, hence, interfered with SN-38 treatment of the cancer cells (*p* < 0.01, Figure 4A). However, the main anthocyanin of BB, Cy3glc, could not be identified as an effective single compound neither at the concentration corresponding to 100 µg/mL BB (15 µM Cy3glc) nor at double the concentration (Figure S5). This is in line with previous results in HT29 cells, where up to 250 µM Cy3glc did not interfere with camptothecin-induced formation of DNA/topo I covalent complexes. However, 10 and 50 µg/mL blackberry extract (76% Cy3glc of total anthocyanins) potently diminished the signal. Furthermore, doxorubicin-induced DNA/topo II α and β signals could be significantly diminished by 50 µg/mL of blackberry extract [14]. Taken together, these results point towards the ability of blackberries to interfere with the therapeutic function of topoisomerase poisons in colon cancer cells. As shown in the cytotoxicity experiments, BB, however, does not seem to interfere with the long-term effects of SN-38.

In non-tumorigenic cells, a different picture was observed. While EB extract seemed to be ineffective in protecting against SN-38-induced toxic effects, BC could significantly prevent respective DNA/topo I intermediate level induction (*p* < 0.01, Figure 4B). Additionally, Bil did not interfere with SN-38 efficacy against cancer cells, but it could protect non-tumorigenic HCEC-1CT cells from SN-38-induced effects, and the signal remained at solvent control level (*p* < 0.001). BB extract, however, clearly interfered with the basal level of covalent complexes present, as a potent decrease in the covalent DNA/topo complexes below solvent control level was evident (## *p* < 0.01, Figure 4B). Again, single anthocyanins were not found to affect the covalent complex levels at the corresponding concentrations (Figure S5B). Even though the anthocyanidin Del and Cy were reported as catalytic inhibitors of topo, reducing the effectiveness of both topo I and II poisons [32], and could therefore interfere with SN-38-mediated response, no effect of the corresponding glycosides Del3glc and Cy3glc was evident at the tested concentrations. It could be hypothesized that the presence of the aglycon is essential for the compound to fit into the binding pocket of topos. However, Webb et al. showed the inhibitory potential of the anthocyanins Cy3glc and Mal3glc in cell free assays at concentrations >50 µM [33]. Hence, at the low concentrations (1.5–15 µM) used in the present study, no effect could be observed. However, combinatory effects with other, unknown compounds of the extracts are plausible.

**Figure 5:**
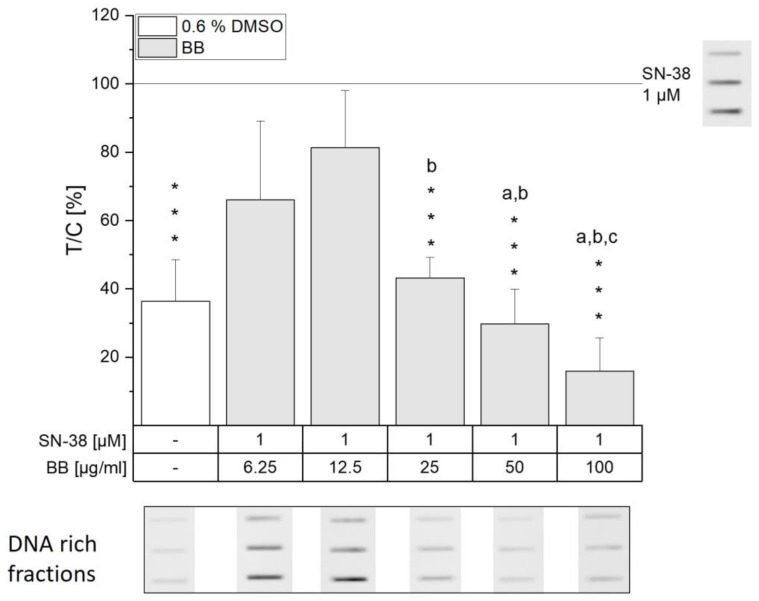
Covalently bound DNA/topo I intermediates after pre-incubation with increasing concentrations of BB extract and subsequent co-incubation with 1 µM SN-38 in HCEC-1CT cells according to the ICE assay. A representative immunoblot from 3-7 biological replicates showing the DNA-rich fractions with equally modified contrast for optimized visualization is shown. Data depicted are the means + SD evaluated as T/C of the SN-38 control in %. Significances compared to the SN-38 control were calculated with one-sample Student’s *t*-tests (*** *p* < 0.001). One-way ANOVA with post hoc Bonferroni test was used to determine statistically significant differences among the tested BB concentrations (*p* < 0.05, a–c; e.g., the letter a represents significance to the lowest concentration of 6.25 µg/mL).

The potent effect on DNA/topo I cleavable complex levels mediated by the BB extract might explain the concentration-dependent toxicity observed in the coupled WST-1 and SRB assay in HCEC-1CT cells (Figure 3 and Figure S3), since crucial cell mechanisms such as transcription or replication would be impaired by the potent interference with topo I function [4,34]. Furthermore, lower doses of BB were found to diminish SN-38-induced covalent complexes in HCEC-1CT cells. Starting from 12.5 µg/mL, a concentration-dependent decrease in DNA/topo I intermediates could be observed, revealing the potency of the BB extract compared to the other extracts tested (Figure 5). The lower concentrations tested thus seemed to protect from SN-38-induced damage to HCEC-1CT cells. However, 100 µg/mL BB was found to significantly diminish covalent complex levels below solvent control, pointing towards potent inhibition of topo I. Whether the BB extract is indeed a potent catalytic inhibitor of topo I leading to the reduction of cleavable complex or a different mechanism is at play remains to be elucidated. Overall, not only the potential collision with the replication fork after topo poisoning but also the excessive interference with the formation of covalent complexes can lead to the induction of DNA strand breaks.

### 3.3. Impact on DNA Damage

**Figure 6:**
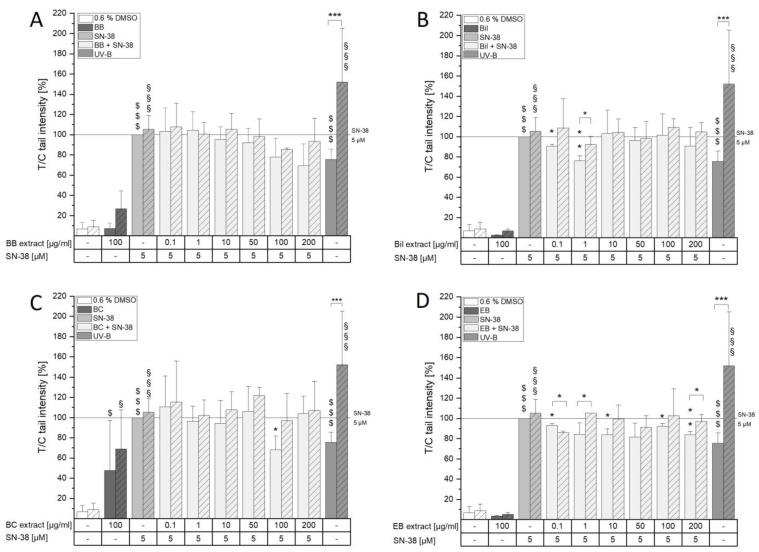
DNA-damaging effects of BB (**A**), Bil (**B**), BC (**C**) and EB (**D**) extracts alone and in combination with SN-38 on HCT116 cells. Cells were exposed to extracts for 30 min and then additionally challenged with 5 µM SN-38 for 1 h. UV-B irradiation for 1 min served as a positive control. Striped bars indicate additional exposure to FPG enzyme. Data shown are the means + SD of 3-4 biological replicates expressed as T/C relative to SN-38 in %. Significant differences to the respective solvent control were calculated with one- and two-sample Student’s *t*-tests and marked with $ (without FPG, $ *p* < 0.05, $$$ *p* < 0.001) and § (with FPG, § *p* < 0.05, §§§ *p* < 0.001). Statistical significances compared to SN-38 were tested with one-sample Student’s *t*-tests (without FPG; * *p* < 0.05, ** *p* < 0.001) or two-sample Student’s *t*-tests (with FPG). Differences between FPG-treatments were calculated with two-sample Student’s *t*-tests and marked with * *p* < 0.05, *** *p* < 0.001.

**Figure 7:**
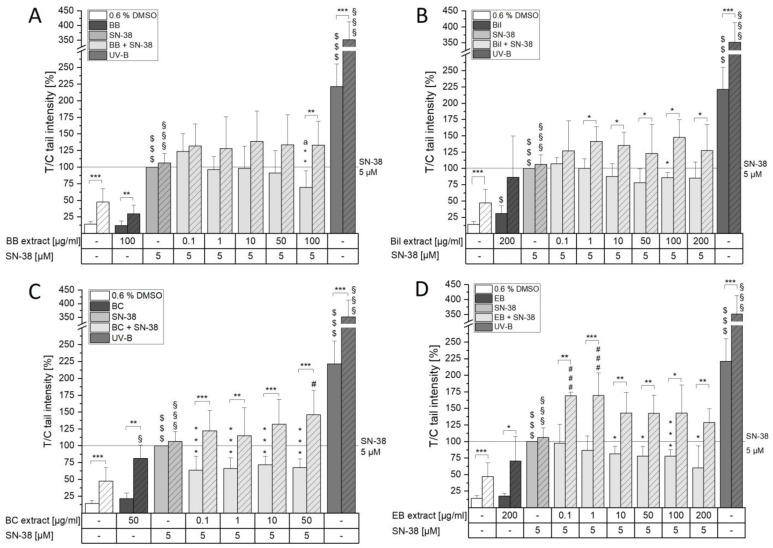
DNA-damaging properties of BB (**A**), Bil (**B**), BC (**C**) and EB (**D**) extracts alone and combined with SN-38 on HCEC-1CT cells. Cells were pre-incubated for 30 min with the extracts and subsequently co-incubated with 5 µM SN-38 for 1 h. Striped bars show an additional treatment with FPG enzyme. As a positive control, 1 min of irradiation with UV-B light was used. Results presented are the means + SD of 3–5 independent replicates evaluated as T/C in % of the SN-38 control. Statistical differences to the respective solvent controls were calculated with one- and two-sample Student’s *t*-tests and marked with $ *p* < 0.05, $$$ *p* < 0.001 (without FPG) or § *p* < 0.05, §§§ *p* < 0.001 (with FPG). Statistical differences compared to SN-38 treated without (* *p* < 0.05, ** *p* < 0.01, *** *p* < 0.001) or with FPG (# *p* < 0.05, ### *p* < 0.001) were calculated with one- or two-sample Student’s *t*-tests, respectively. One-way ANOVA with post hoc Bonferroni test was used to calculate differences among the tested concentrations (*p* < 0.05, a). Differences between FPG treatments were calculated with two-sample Student’s *t*-tests and marked with * *p* < 0.05, ** *p* < 0.01, *** *p* < 0.001.

After stabilization of the covalent complex by SN-38, a higher DNA strand break rate is expected since not only tension on the DNA strands is unreleased but also collisions of the DNA/topo intermediate with the replication fork can occur [4]. It was, thus, of great interest to determine whether anthocyanin-rich extracts could interfere with SN-38-induced DNA damage and how this might be related to their effect on topoisomerases. HCT116 and HCEC-1CT cells were pre-incubated for 30 min with the extracts and then additionally challenged with 5 µM SN-38 for 60 min. The concentration of SN-38 was chosen from a tested concentration range of 0.1–10 µM SN-38 in both cell lines (Figure S6). With a tail intensity of 23.5 ± 4.5%, 5 µM SN-38 led to more DNA damage in HCT116 cells compared to HCEC-1CT cells (13.2 ± 1.9%, Figure S6). To simplify comparison of effects between the cell lines, tail intensity results were expressed relative to 5 µM SN-38. Out of the extracts, solely 100 µg/mL BC induced tail intensity of HCT116 cells significantly, indicating DNA-damaging properties of the extract (Figure 6C). In HCEC-1CT cells, BC led to DNA damage induction only when additionally treated with formamidopyrimidine-DNA-glycosylase (FPG) (Figure 7C), while 100 µg/mL Bil did so in the non-FPG-treated sample and showed a non-significant tendency in FPG-treated cells (Figure 7B). Of note, the potent cytotoxic effects of BB are apparently not the result of DNA damage induction since no difference to the control could be observed in either cell line (Figure 6A and Figure 7A).

Compared to the solvent control, SN-38 as well as the positive control (irradiation with UV-B light) could significantly induce DNA damage in all replicates in both cell lines tested (Figure 6 and Figure 7). Additionally, cells treated with UV-B light and FPG showed significantly higher tail intensity than non-FPG-treated cells. However, SN-38 did not induce additional oxidative damage in both cell lines since no difference between FPG and non-FPG-treated cells could be observed. This was expected to some extent, since the special feature of camptothecin-derived drugs is their target selectivity for topo I, making topo I poisoning the sole direct mechanism of action [35,36]. However, potential secondary mechanisms resulting in consequence of topo I poisoning, such as DNA damage, oxidative stress, etc., should not be neglected. Overall, the extracts diminished the SN-38-induced effect in HCT116 cells at different concentrations. BB showed concentration-dependent tendencies to reduce the signal. At 100 µg/mL, however, the non-significant reduction did not resemble the potent effect observed on DNA/topo I levels (Figure 4A). Nonetheless, a higher concentration of SN-38 was used in the comet assay compared to the ICE assay, and BB might, hence, not be able to prevent SN-38-mediated signal induction to such a great extent. Bil reduced DNA damage at the lowest concentrations tested (0.1–1 µg/mL) but not at higher concentrations. Furthermore, 100 µg/mL BC not only induced DNA damage on its own but also diminished SN-38-induced tail intensity significantly, hinting further towards BC hindering SN-38 efficacy in HCT-116 cells, as was already shown in tendencies on DNA/topo I levels (Figure 4A). EB showed reducing effects over the whole concentration range tested. However, none of the extracts concomitantly affected FPG-sensitive sites (Figure 6), which could hint towards oxidative damage by the extracts.

In HCEC-1CT cells, cytotoxic properties of BB and BC only allowed for testing up to 100 and 50 µg/mL, respectively. BC, nonetheless, reduced SN-38-induced DNA damage potently over the whole concentration range. While only 100 µg/mL Bil could diminish SN-38-induced DNA damage, a concentration-dependent reduction by EB could be observed starting from 1 µg/mL (Figure 7D). BB only diminished SN-38-mediated tail intensity at 100 µg/mL and did not follow the potent concentration-dependent effects observed in the ICE assay (Figure 5). However, if topoisomerase function is impaired to the extent that was observed in the ICE assay, normal cell function cannot be guaranteed, and a higher tail intensity from unrelieved torsion tension could be the result. Despite the protective tendencies of the extracts in the non-tumorigenic colon cells, no reducing effect on FPG-sensitive sites could be observed. On the contrary, the extracts seem to even further induce the FPG-dependent signals, hinting towards an implication with oxidative stress.

Overall, our experiments showed comparable results in the tumorigenic and non-tumorigenic cell lines with slightly more pronounced effects in HCEC-1CT cells. To the best of our knowledge, the available literature on potential protective effects of anthocyanin-rich extracts on chemotherapy-induced damage was so far only obtained in cancer cells, and no comparison to healthy cells had been conducted before. A bilberry and a blackberry extract were shown to reduce camptothecin-induced DNA damage in a concentration-dependent manner starting from 1 µg/mL in HT29 cells. Furthermore, the strand-breaking activity induced by topo II poison doxorubicin could be diminished by these extracts and a red grape extract in HT29 cells. A slight reducing tendency for the anthocyanin Cy3glc could also be determined against both camptothecin and doxorubicin [14,37]. However, a concentration of 100 µM by far exceeds what can be found in realistic extract concentrations. Furthermore, no reducing effect of 100 µM Cy3glc could be observed against the more potent topo I poison SN-38 in murine CT26 cells. Comparable to the presented data in HCT116 cells, the anthocyanin-rich extracts slightly reduced the SN-38-induced DNA damage in CT26 cells without concomitant effects on FPG-sensitive sites [15]. Since the purpose of chemotherapeutic drugs is clearly to harm cancer cells, any effects of anthocyanins and extracts reducing their effects in cancer cells should be interpreted with care. A crucial factor for further evaluation of the combinatory effects would be the localization of the tumor site. Primary tumors localized in the GIT would, thus, be exposed to both the drug and the anthocyanins or extracts consumed via the diet, whereas secondary tumors, spread to a different part of the body, would be facing less of the naturally bioactive substances, since systemic bioavailability is reported to be low [38]. In the latter case, it might therefore be speculated that anthocyanins exhibit eventual beneficial properties in protecting healthy cells of the GIT from adverse effects of chemotherapeutic treatment without interference with the successful treatment of the cancer if sufficient systemic bioavailability is necessary to reach the tumor tissue. For instance, most of the literature reports that less than 1% of anthocyanins are bioavailable, and bioavailability of anthocyanins is dependent on aglycone structure as well as the attached sugar moiety [38]. Furthermore, peak concentrations in plasma after anthocyanin consumption are reported between 30 min and 2 h, demonstrating that anthocyanins are absorbed and excreted rather rapidly [39]. However, the impairment of the intestinal barrier as a consequence of on-going chemotherapy [40] could modulate absorption patterns of anthocyanins from the GIT of cancer patients and has to be further investigated to fully understand the bioavailability of anthocyanins and the potential interference at a secondary tumor site.

### 3.4. Oxidative Properties of Berry Extracts

**Figure 8:**
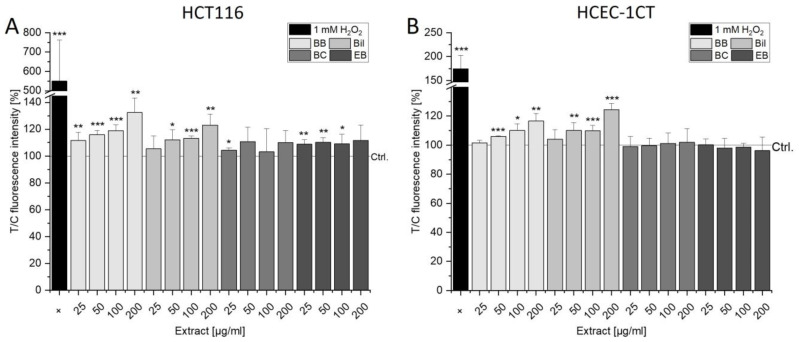
ROS-dependent induction of oxidative stress in HCT116 (**A**) and HCEC-1CT (**B**) cells after 30 min of exposure to anthocyanin-rich extracts measured within the DCF assay. Cells were stained with DCFH-DA solution for 15 min and afterwards incubated with the extracts for 30 min. Results shown are the means + SD of 4–6 independent experiments evaluated as T/C of the fluorescence intensity signal produced by the incubation solutions in % relative to the solvent control (0.5% DMSO). For verification of signal induction, 1 mM H_2_O_2_ was used as a positive control. Significant differences to the control were determined with one-sample Student’s *t*-tests (* *p* < 0.05, ** *p* < 0.01, *** *p* < 0.001).

**Figure 9:**
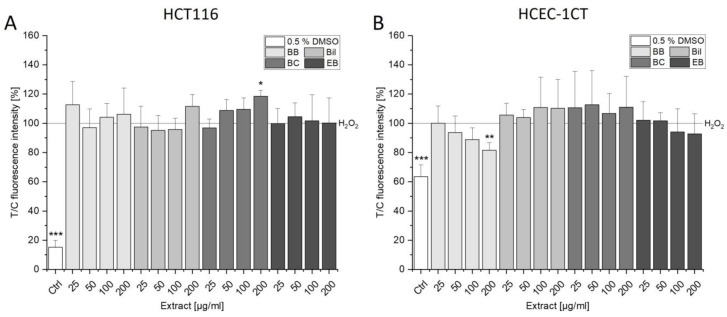
Effect of anthocyanin-rich extracts on H_2_O_2_-dependent ROS induction in HCT116 (**A**) and HCEC-1CT (**B**) cells after 30 min. Cells were pre-incubated for 24 h with extracts, stained with DCFH-DA solution for 15 min and subsequently challenged with 1 mM H_2_O_2_. Results presented are the means + SD of 4–6 biological replicates evaluated as T/C of the fluorescence intensity signal relative to the H_2_O_2_ control after 30 min. Cells treated only with 0.5% DMSO were used as control for proper signal induction by H_2_O_2_. Significant differences to the H_2_O_2_ control were calculated with one-sample Student’s *t*-tests (* *p* < 0.05, ** *p* < 0.01, *** *p* < 0.001).

A vast amount of the literature describes the potent anti-oxidative properties of anthocyanins and points towards their potential chemopreventive action. However, the comet assay pointed towards induction of oxidative damage by the extracts in FPG-treated samples (Figure 6 and Figure 7). The DCF assay was, therefore, performed to assess the potential of the applied anthocyanin-rich extracts to induce oxidative damage. As expected from the comet assay, where SN-38 did not affect the level of FPG-sensitive sites in both cell lines, SN-38 could not be used as a positive control. No potent induction of the signal was observed when a concentration range of 1–10 µM was tested in the DCF assay (Figure S7). In colon26 cells, however, 5–50 µM SN-38 concentration-dependently induced oxidative stress after 60 min [41], pointing out the necessity of testing several cell lines to ameliorate interpretation of potential secondary mechanisms of drugs. Hence, in this study, H_2_O_2_ was used as a positive control in the DCF assay (Figure 8) and as an oxidative stressor in the pDCF assay (Figure 9). Over time, signals of the positive control were increasing in both cell lines (Figure S8). However, this was to a lesser extent in HCEC-1CT cells than in HCT116 cells, hinting towards HCEC-1CT cells better coping with oxidative stress since a higher base level of oxidative stress is expected for cancer cells. BB induced oxidative stress in both cell lines concentration-dependently (Figure 8), and 200 µg/mL BB showed slight time-dependent tendencies in HCT116 cells but not in HCEC-1CT cells (Figure S8A,B). After 30 min of incubation, Bil also induced oxidative stress in both cell lines from 25–200 µg/mL. While BC did not induce oxidative stress and mostly remained at solvent control levels, 25–100 µg/mL EB led to an induction of oxidative stress in HCT116 cells.

Interestingly, in HCEC-1CT cells, incubation with EB significantly decreased the signal after longer exposure (60 and 90 min, Figure S8H) but not after 30 min (Figure 8B). However, the decrease was only time- but not concentration-dependent as higher concentrations did not lead to more potent effects. Since both BB and Bil exerted pro-oxidative properties in the DCF assay, their single main anthocyanins were tested. Comparable to the available literature, Cy3glc showed significant anti-oxidative properties starting from a concentration of 15 µM (equivalent to conc. in 100 µg/mL BB) in both cell lines (Figure S9). In Caco-2 cells, it was shown that Cy3glc and other cyanidin-derived anthocyanins exhibited anti-oxidative potential starting from 1 µM [21]. Del3glc, conversely, was ineffective in the tested concentrations, and signal intensity remained at solvent control level. This effect is backed up by the literature, since Del3glc was only found to significantly reduce porcine aortic endothelial cell redox status at concentrations higher than 80 µM [42].

In contrast to the classical DCF assay, in the protective DCF (pDCF) assay, the cells are incubated for 24 h to allow an impact on the transcription and translation of proteins and enzymes involved in oxidative defense, such as glutathione, hem oxygenase 1 or glutamate-cysteine ligase [21] prior to exposure to H_2_O_2_. Lower signals, hence, indicate an anti-oxidative effect since induced oxidative stress is counteracted. However, in HCT116 cells, none of the extracts showed respective anti-oxidative properties. Conversely, 200 µg/mL BC even further induced the oxidative stress signal (Figure 9A). This inducing effect was also observed with 50 and 100 µg/mL BC at longer measurement times (60 and 90 min) and can generally be described as time-dependent induction since no effects occurred at 0–15 min (Figure S10E). In HCEC-1CT cells, contrarily, BB was found to exert concentration-dependent anti-oxidative effects reaching significance at 200 µg/mL (Figure 9B). This effect was not the consequence of cytotoxicity, since an SRB assay after 24 h revealed that none of the extracts induced cytotoxic effects in HCEC-1CT cells. Additionally, this anti-oxidative effect could only be observed at 30 and 45 min, since at 200 µg/mL, a slightly U-shaped curve was obtained (Figure S10B). Cy3glc and Cy3sam were described in the literature for their anti-oxidative properties in Caco-2 cells. However, significant effects could only be demonstrated for concentrations ≥100 µM [21], which exceeds the corresponding amount of Cy3glc in the BB extract more than three-fold. Hence, Cy3glc might contribute to the anti-oxidative effect by 200 µg/mL BB, but combinatory effects with other anti-oxidative compounds comprising the extract have to be considered.

### 3.5. Influence on the NF-κB Pathway and Inflammation

A variety of transcription factors can be activated by oxidative stress signals and lead to inflammation [43]. NF-κB is among these transcription factors, and its activation by the anthocyanin-rich extracts was investigated in THP1-Lucia^TM^ monocytes. Upon initiation of the pathway, Lucia^TM^ luciferase is expressed proportionally to the activation. As a first step, the effect of SN-38 on the NF-κB pathway was investigated with and without additional stimulation with LPS. At first glance, SN-38 appeared to potently diminish both LPS-stimulated NF-κB activation and the non-LPS stimulated NF-κB signal at 0.5–10 µM. However, these reducing effects were attributable to the potent cytotoxicity of SN-38 in THP1-Lucia^TM^ cells since a concentration-dependent reduction of cell viability was evident in the CTB assay starting from 100 nM (Figure S11C,D). Hence, overall, SN-38 did not influence the NF-κB pathway in the cell model tested. The available literature on NF-κB pathway activation by SN-38 is inconclusive. On the one hand, SN-38 was reported to potently activate the NF-κB pathway in colon26 [41], HeLa [44], NCI-N87 and AGS human gastric cancer cells [45]. Furthermore, SN-38 could increase LPS-stimulated NF-κB activation in THP1 cells [46]. On the other hand, Wong et al. reported a reduction in LPS-induced NF-κB activation in RAW 264.7-Luc macrophages when exposed to 0.2–20 µM SN-38 for 6 h. However, no effect other than cytotoxicity could be shown on non-stimulated macrophages [47], comparable to the results presented here in THP1-Lucia^TM^ monocytes.

**Figure 10:**
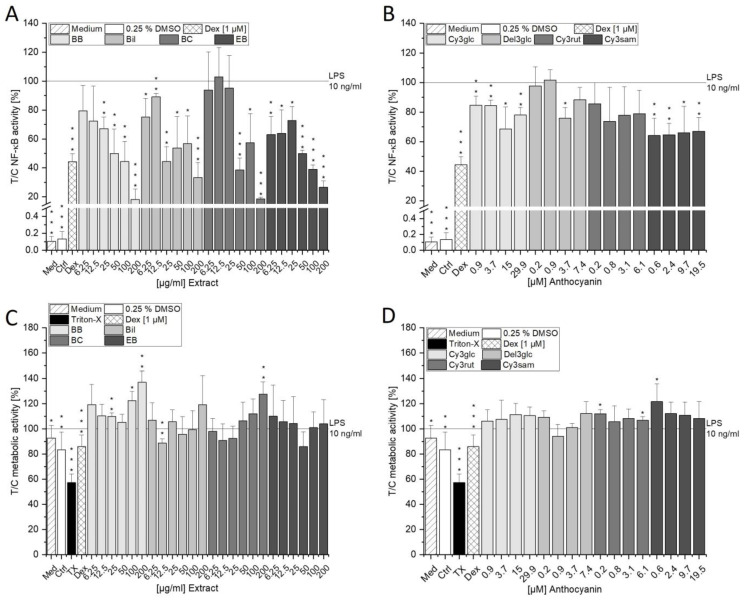
Immunomodulatory effects of anthocyanin-rich extracts (**A**) and single anthocyanins (**B**) and their corresponding cytotoxic properties (**C**,**D**) measured with the NF-κB and CTB assay, respectively. THP1-Lucia^TM^ cells were pre-incubated with extracts or anthocyanins for 2 h and subsequently additionally challenged with 10 ng/mL LPS for 18 h. Pre-incubation with 1 µM Dex served as positive control for anti-inflammatory properties. Cell culture medium and 0.25% DMSO served as controls for proper activation of the NF-κB pathway. For the CTB cytotoxicity assay, 0.01% TX served as a positive control. Results show the mean + SD of 3-5 independent replicates measured in technical triplicates expressed as T/C in % relative to the LPS control. Statistically significant differences to the LPS control were calculated with one-sample Student’s *t*-tests (* *p* < 0.05, ** *p* < 0.01, *** *p* < 0.001).

Since SN-38 could not be used to induce the NF-κB pathway in the presented cell model, 10 ng/mL LPS was utilized instead, which led to a potent induction of the signal in all experiments performed. The four berry extracts significantly prevented the activation of the NF-κB pathway. BB showed concentration-dependent effects with 100 µg/mL at the same effect level as 1 µM of the anti-inflammatory drug dexamethasone (Figure 10A). The main anthocyanin of BB, Cy3glc, diminished the signal significantly at all concentrations tested and might, therefore, contribute to the effect of BB in combination with other extract components (Figure 10B). In THP1-Lucia^TM^ macrophages (differentiated from the herein-used monocytes), 10 µM Cy3glc could only marginally reduce LPS-induced NF-κB activation [48], pointing towards a discrepancy of macrophages and monocytes in their reactiveness. In contrast, another study conducted by Di Gesso et al. found 10 µM Cy3glc to be ineffective in THP1 monocytes [49]. However, a higher LPS concentration (100 ng/mL) was used, and pre-incubation with Cy3glc was only performed for 30 min [49], which could dampen the weak anti-inflammatory effect of Cy3glc observed in the present study. While 100 mg/mL of blackberry extract led to a potent inhibition of LPS-induced NF-κB activation in U937-3χκB-LUC human monocytes, 30 mg/mL black currant extract further induced the luciferase activity [50]. In the present study, BC, conversely, potently diminished luciferase expression at 50–200 µg/mL. Furthermore, Bil as well as EB inhibited LPS-induced NF-κB pathway activation over the whole concentration range, with the highest concentrations reaching 33.3% and 26.5%, respectively. Overall, the observed immunosuppressive effects could not be attributed to cytotoxicity since cell viability was not diminished in the respective concentration ranges (Figure 10C,D). On the contrary, at 200 µg/mL of BB and BC, the fluorescence signal was induced to 130–140% of the control, hinting towards an induction of metabolic stress by these extracts. It can, thus, not be excluded that potential metabolic stress influenced the effects on NF-κB activity. The single anthocyanin Cy3rut only showed non-significant tendencies to reduce NF-κB activation (Figure 10B). In previous studies, up to 50 µM of Cy3rut failed to potently diminish LPS-induced NF-κB activation in A549 cells. Only the highest concentration of 100 µM Cy3rut could reduce NF-κB protein as shown in Western blot experiments [51], pointing towards the low anti-inflammatory potential of Cy3rut. In the present study, Del3glc inhibited NF-κB activation significantly at 3.7 µM but failed to do so in the highest tested concentration of 7.4 µM. To the best of our knowledge, we are the first to report the inhibitory potential of Cy3sam on LPS-induced NF-κB pathway activation. So far, anti-inflammatory properties have only been reported for 50–200 µM delphinidin-3*O*-sambubioside in RAW264.7 cells [52]. However, we could show that Cy3sam exhibits its anti-inflammatory properties in much lower concentrations of 0.6–19.5 µM in THP-1 Lucia monocytes.

Since investigations of the NF-κB pathway activation showed strong indications that the anthocyanin-rich berry extracts act as anti-inflammatory agents, qRT-PCR was performed in both tumorigenic HCT116 and non-tumorigenic HCEC-1CT cells to assess the effect on pro-inflammatory cytokine transcription. After pre-incubation for 2 h with the extracts, cells were co-incubated for 3 h with 25 ng/mL IL-1β protein to induce the NF-κB pathway and, hence, the transcription of pro-inflammatory cytokines IL-1β, IL-8, TNF-α and COX-2. Interestingly, no reduction in IL-1β-induced inflammation could be observed. On the contrary, tendencies to further increase the inflammatory response were observed. For instance, Bil showed further inductive potential on IL-1β-induced transcription of IL-1β and TNF-α in HCT116 cells as well as IL-8 and COX-2 in HCEC-1CT cells, yet not reaching significance (Figure S12). While BC and EB also tended to further increase TNF-α expression in HCT116, 100 µg/mL BB significantly induced (*p* < 0.05) COX-2 transcription.

This stands in contrast to the available literature in colorectal cancer cells where blackberry [48,53,54], bilberry [55], elderberry [56] and black currant [57] extracts have been reported to act as anti-inflammatory agents. However, the composition and concentration of individual compounds naturally differs among extracts, potentially interfering with anti-inflammatory effects on selected cytokines. Additionally, time-points and inflammatory stimuli could have affected the outcome. Taken together, the present study indicates a different immunomodulatory response to berry-derived polyphenols locally in the GIT versus blood-stream-related effects. With both tumorigenic and non-tumorigenic colon cells, pro-inflammatory effects appear to dominate, whereas for monocytes, representing the blood compartment, substantial anti-inflammatory activity was identified. Of course, the systemic bioavailability of the different extract constituents needs to be considered, never expecting the whole berry extract to reach the blood stream. Nevertheless, anti-inflammatory activity of selected single anthocyanins was already observed in sub-micromolar concentrations. Thus, systemic immunomodulatory effects cannot totally be excluded.

## 4. Conclusions

Overall, this study showed that anthocyanin-rich extract concentrations cytotoxic to cancerous cells might also harm non-tumorigenic cells. Healthy intestinal tissue is prone to interfere with these bioactive food ingredients, whereas cancer cells—depending on whether they are part of primary or secondary colon cancer—might not even be exposed to toxic polyphenol concentrations. A potent, concentration-dependent cytotoxicity of the blackberry extract that persisted in combination with SN-38, further pronouncing the cytotoxic properties mediated by the anti-cancer drug in both cell lines, was uncovered. The potent inhibition of DNA/topoisomerase I covalent complexes and the induction of oxidative stress could be unraveled as potential underlying mechanisms for BB toxicity. Furthermore, observed effects were not solely attributable to single key anthocyanins and are presumably the result of mixture effects with other bioactive compounds comprising the polyphenol extracts. Further analysis of non-anthocyanin extract compounds and investigations on mixture effects of these could shed light on bioactive compounds in extracts. Altogether, local high doses of natural bioactives—as found in respective food supplements—can also mediate unwanted effects, and undesirable mixture effects in combination with chemotherapy should be taken into consideration.

## Figures and Tables

**Table 1:** Concentration of Cy3glc, Del3glc, Cy3rut and Cy3sam in µM corresponding to extract concentrations in µg/mL (e.g., 200 µg/mL BB extract contains 29.9 µM Cy3glc).

	[µM]			
Extract [µg/mL]	Cy3glc in BB	Del3glc in Bil	Cy3rut in BC	Cy3sam in EB
200	29.9	7.4	6.1	19.5
100	15.0	3.7	3.1	9.7
50	7.5	1.8	1.5	4.9
25	3.7	0.9	0.8	2.4
12.5	1.9	0.5	0.4	1.2
6.25	0.9	0.2	0.2	0.6

## Data Availability

Data is contained within the article and Supplementary Materials.

## Supplementary Materials

The following supporting information can be downloaded at: https://www.mdpi.com/article/10.3390/antiox13070846/s1, Figure S1: Chemical structures of anthocyanidins and sugars bound to position 3 of anthocyanidins. Figure S2: Cytotoxicity of BB, Bil, BC and EB extracts in HCT116 cells measured with SRB assay. Figure S3: Cytotoxicity of BB, Bil, BC and EB extracts in HCEC-1CT cells measured with SRB assay. Figure S4: Cytotoxicity of Cy3glc, Del3glc, Cy3rut and Cy3sam in HCT116 and HCEC-1CT cells measured with SRB assay. Figure S5: ICE assay detection of covalently bound DNA/topo I intermediates in HCT116 and HCEC-1CT cells exposed to single anthocyanins. Figure S6: DNA-damaging properties of SN-38 in HCT116 and HCEC-1CT. Figure S7: ROS-dependent induction of oxidative stress in HCT116 and HCEC-1CT cells after exposure to SN-38. Figure S8: ROS-dependent induction of oxidative stress in HCT116 and HCEC-1CT cells after exposure to BB, Bil, BC and EB. Figure S9: ROS-dependent induction of oxidative stress in HCT116 and HCEC-1CT cells after exposure to Cy3glc and Del3glc. Figure S10: Effect of anthocyanin-rich extracts on H_2_O_2_-dependent ROS induction in HCT116 and HCEC-1CT cells. Figure S11: Immunomodulatory and cytotoxic effects of SN-38 with and without additional stimulation with LPS. Figure S12: Relative gene transcription of IL-1β, IL-8, TNF-α or COX-2 in HCT116 and HCEC-1CT cells.
