# Supplementary material for: Anthocyanin-Rich Berry Extracts Affect SN-38-Induced Response: A Comparison of Non-Tumorigenic HCEC-1CT and HCT116 Colon Carcinoma Cells
Source: Antioxidants (Basel). 2024 Jul 15;13(7):846. doi: 10.3390/antiox13070846 (PMC11273996; doi:10.3390/antiox13070846)
Supplement: Supplementary file 1 [file antioxidants-13-00846-s001.zip › antioxidants-3093577-supplementary.pdf]

Supplementary information

Anthocyanin-rich berry extracts affect SN-38 induced response: a comparison of non-tumorigenic HCEC-1CT and HCT116 colon carcinoma cells

Cornelia Schmutz[1,2], Crepelle Plaza[1], Franziska Steiger[1], Natascha Stoirer[1], Judith Gufler[1,2], Gudrun Pahlke[1], Frank Will[3], Walter Berger[4], Doris Marko[1,*]

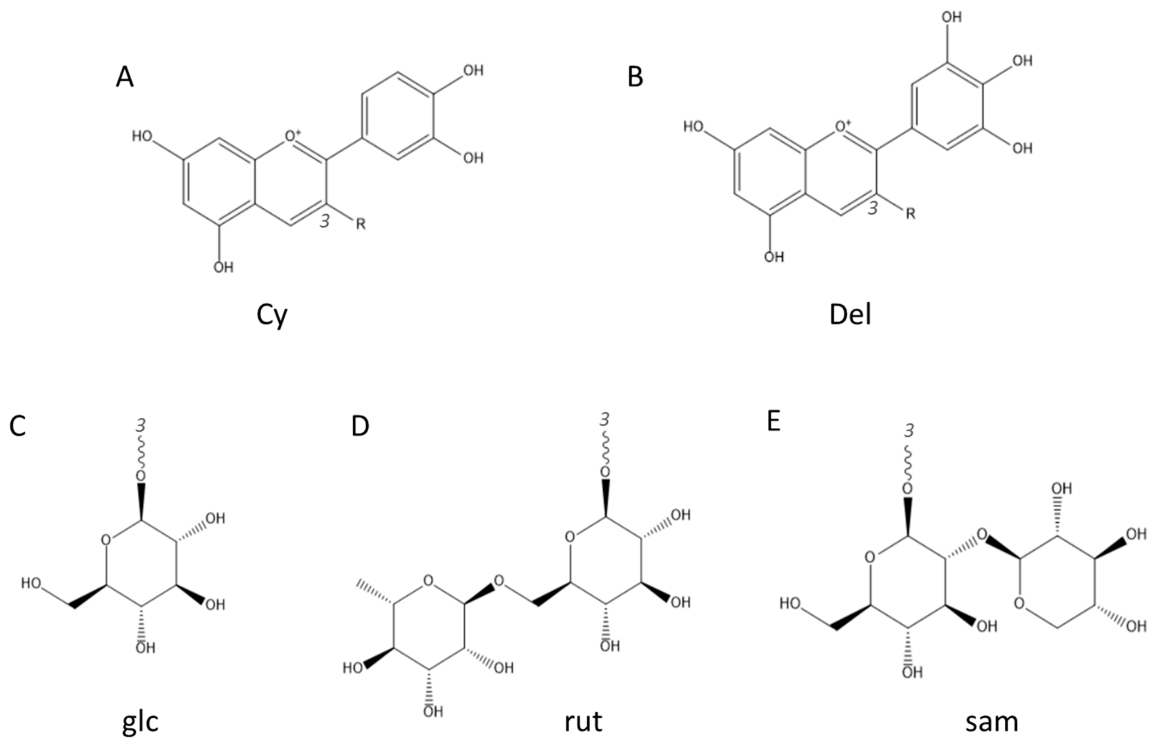

Figure S1: Chemical structures of anthocyanidins cyanidin (**A**, R=OH, Cy) and delphinidin (B, R=OH, Del) and the sugars bound to position 3 of the anthocyanidins glucoside (C, glc), rutinoside (D, rut) and sambubioside (E, sam).

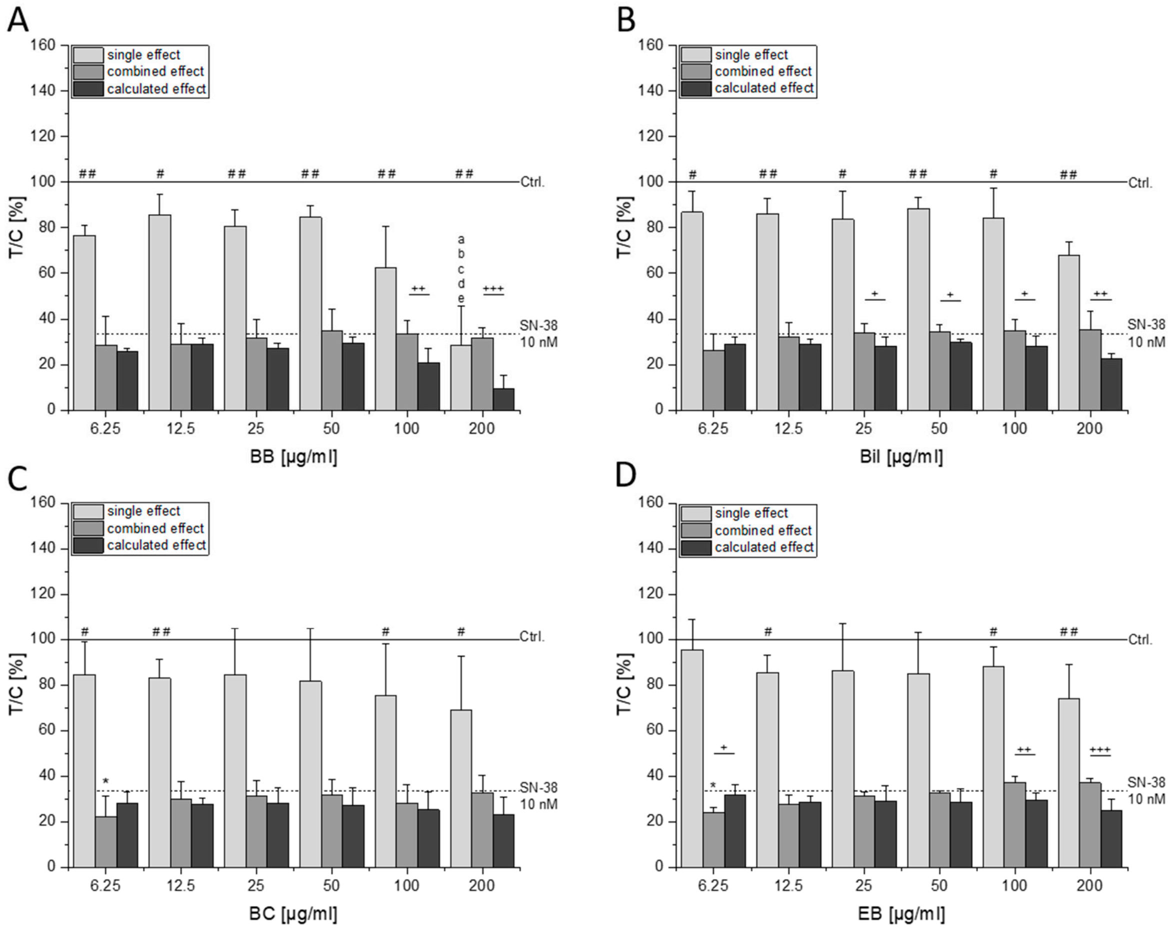

Figure S2: Cytotoxicity of BB (**A**), Bil (**B**), BC (**C**) and EB (**D**) extracts in HCT116 cells after 72 h of exposure measured with SRB assay. Results are depicted as mean + SD of 3-5 biologically independent replicates relative to the solvent control (Ctrl., 0.6% DMSO). The dashed line indicates the mean value received after 72 h incubation with 10 nM SN-38 (33.5 ±8.4%). Cells were either incubated with extract alone (light grey bars) or together with 10 nM SN-38 (middle grey bars) and statistical differences to the controls were tested with one-sample (# p<0.05, ## p<0.01) or two-sample (* p<0.05) Student's *t*-test, respectively. One-way ANOVA with post-hoc Bonferroni test was used to calculate significant differences among tested concentrations (p<0.05, a-e). Furthermore, the expected additive effect of their combination (dark grey bars) was calculated using the model of IJA and significant differences according to two-sample Student's *t*-test were marked with + p<0.05, ++ p<0.01, +++ p<0.001.

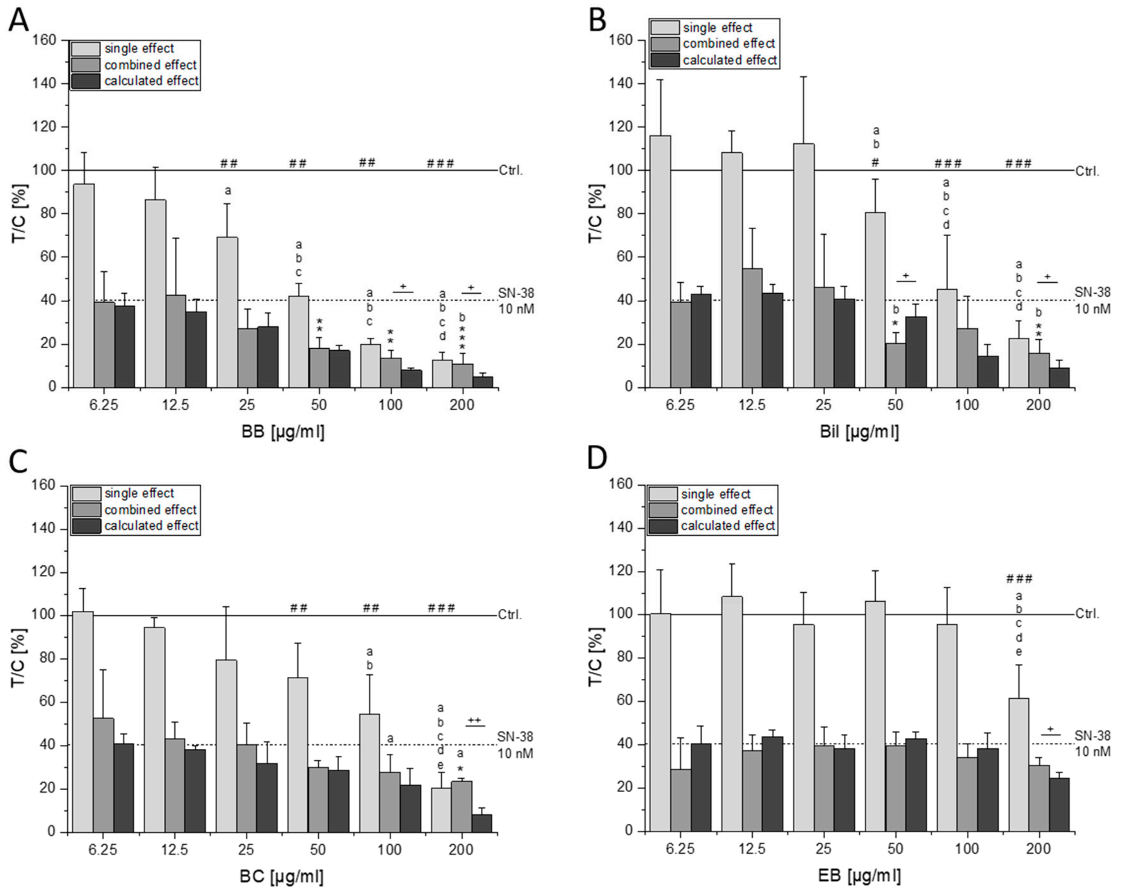

Figure S3: Cytotoxicity of BB (**A**), Bil (**B**), BC (**C**) and EB (**D**) extracts in HCEC-1CT cells after 72 h of exposure measured with SRB assay. Results are depicted as mean + SD of 3-5 biologically independent replicates relative to the solvent control (Ctrl., 0.6% DMSO). The dashed line indicates the mean value received after 72 h incubation with 10 nM SN-38 (40.2 ±15.3%). Cells were either incubated with extract alone (light grey bars) or together with 10 nM SN-38 (middle grey bars) and statistical differences to the controls were tested with one-sample (## p<0.01, ### p<0.001) or two-sample (* p<0.05, ** p<0.01, *** p<0.001) Student's *t*-test, respectively. One-way ANOVA with post-hoc Bonferroni test was used to calculate significant differences among tested concentrations (p<0.05, a-e). Furthermore, the expected additive effect of their combination (dark grey bars) was calculated using the model of IJA and significant differences according to two-sample Student's *t*-test were marked with + p<0.05, ++ p<0.01.

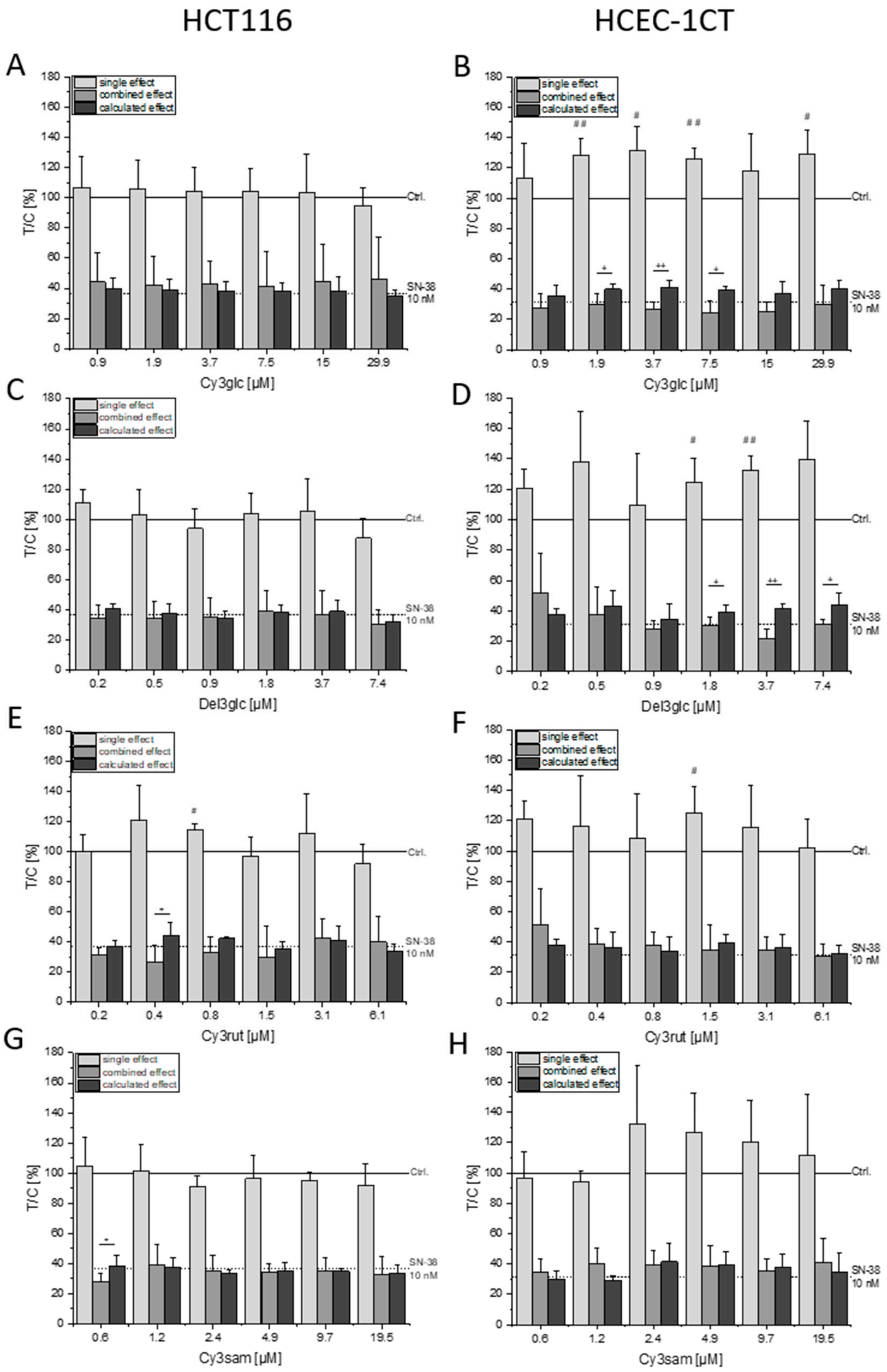

Figure S4: Cytotoxicity of Cy3glc (**A, B**), Del3glc (**C, D**), Cy3rut (**E, F**) and Cy3sam (**G, H**) in HCT116 (left column) and HCEC-1CT (right column) cells after 72 h of exposure measured with SRB assay. Results are depicted as mean + SD of 3-5 biologically independent replicates relative to the solvent control (Ctrl., 0.6% DMSO). Cells were either incubated with anthocyanin alone (light grey bars) or together with 10 nM SN-38

(middle grey bars) and statistical differences to the controls were tested with one-sample (# p<0.05, ## p<0.01) or two-sample Student's *t*-test, respectively. The dashed line indicates the mean value received after 72 h incubation with 10 nM SN-38 (36.7 ±12.5% for HCT116, 31.3 ±13.0% for HCEC-1CT). Furthermore, the expected additive effect of their combination (dark grey bars) was calculated using the model of IJA and significant differences according to two-sample Student's *t*-test were marked with + p<0.05, ++ p<0.01.

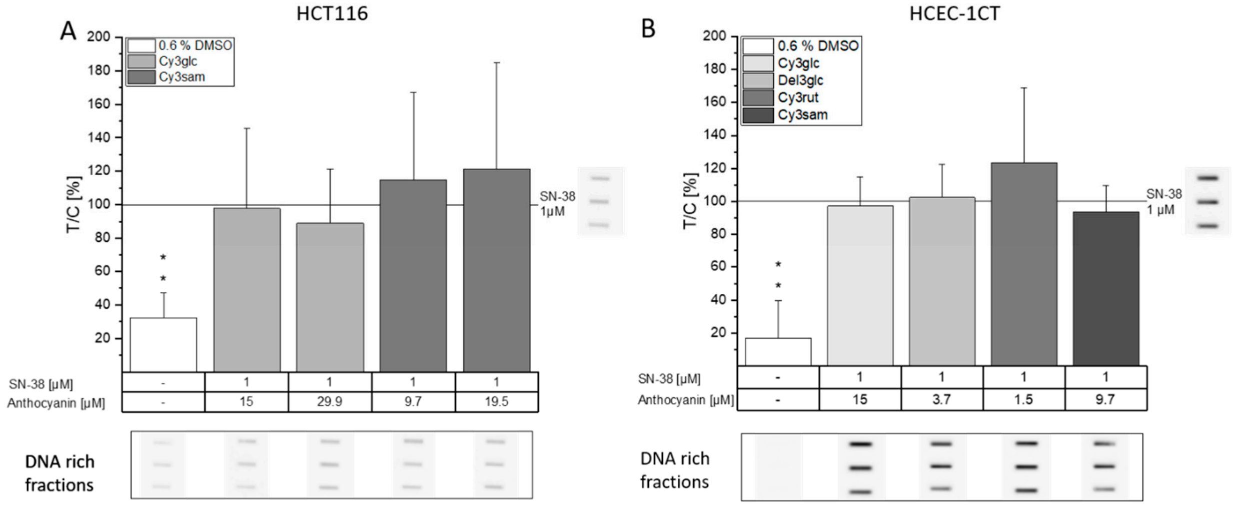

Figure S5: ICE assay detection of covalently bound DNA/topo I intermediates in HCT116 (**A**) and HCEC-1CT (**B**) cells. Cells were pre-incubated with anthocyanins for 30 min, followed by co-incubation with 1 µM SN-38 for 1 h. A representative immunoblot from 3-5 biological replicates depicting the DNA rich fractions is shown with equally modified contrast to improve visualization. Data shown are the means + SD evaluated as T/C of the SN-38 control in %. Significances compared to the SN-38 control were calculated with one-sample Student's t-test (** p<0.01).

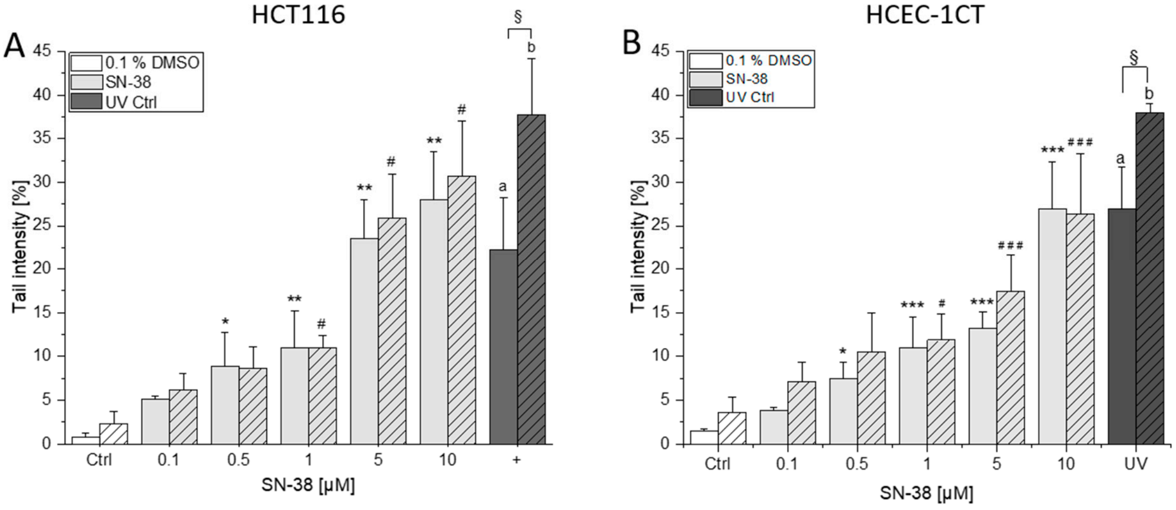

Figure S6: DNA-damaging properties of increasing concentrations of SN-38 in HCT116 (**A**) and HCEC-1CT (**B**) cells assessed with the comet assay. Cells were incubated for 1 h and irradiation with UV-B light for 1 min served as positive control. Striped bars indicate additional treatment with FPG enzyme. Data shown is the mean + SD of 4-5 independent replicates evaluated as tail intensity in %. Statistically significant differences to the respective DMSO control were determined with one-way ANOVA (without FPG: * p<0.05, ** p<0.01, *** p<0.001; with FPG: # p<0.05, ### p<0.001) or two-sample Student's *t*-test (p<0.001, a without FPG, b with FPG). Differences between FPG treatments were calculated with two-sample Student's *t*-test and marked with § (p<0.05).

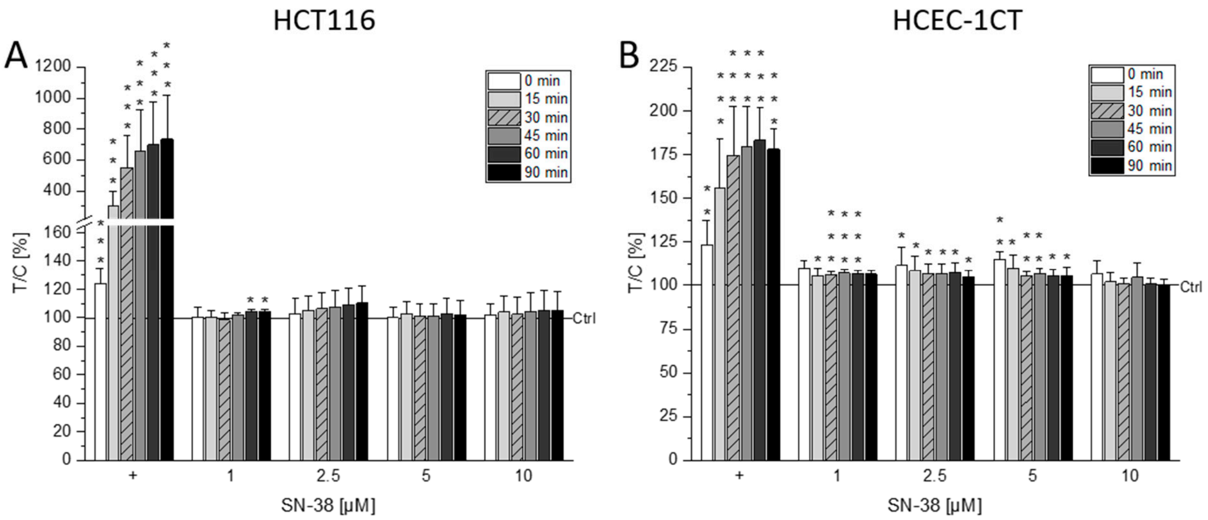

Figure S7: ROS-dependent induction of oxidative stress in HCT116 (**A**) and HCEC-1CT (**B**) cells after exposure to SN-38 from 0-90 min measured with the DCF assay. Cells were stained with DCFH-DA solution for 15 min and afterwards incubated with increasing concentrations of SN-38. 1 mM H_2O_2 was used as a positive control for verification of signal induction. Data presented is the mean + SD of 5-6 independent measurements expressed as T/C of the fluorescence signal relative to the solvent control (Ctrl, 0.1% DMSO). Significant differences to the control were determined with one-sample Student's *t*-test (* p<0.05, ** p<0.01, *** p<0.001).

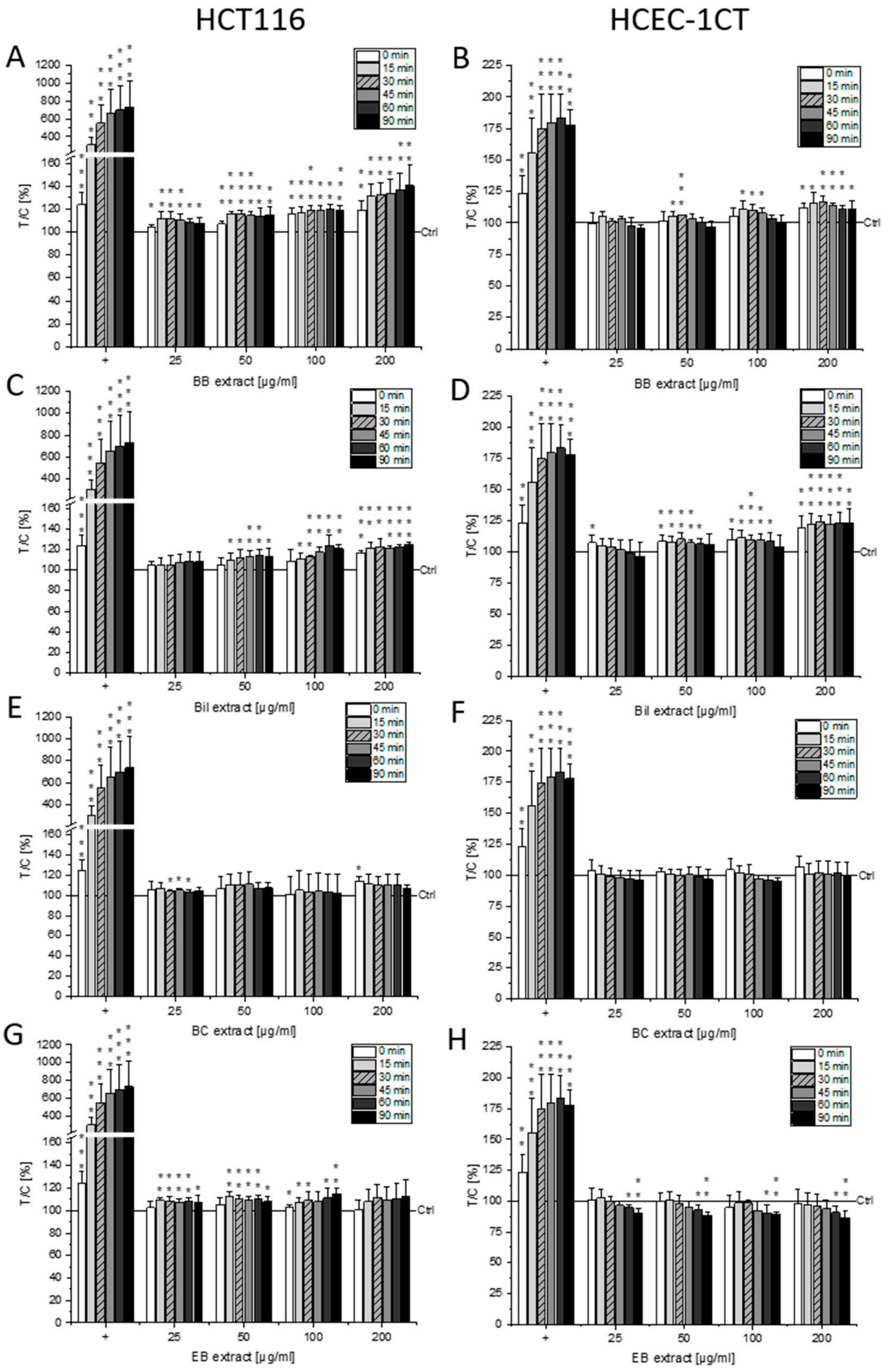

Figure S8: ROS-dependent induction of oxidative stress in HCT116 (left) and HCEC-1CT (right) cells after exposure to BB (**A, B**), Bil (**C, D**), BC (**E, F**) and EB (**G, H**) from 0-90 min measured with the DCF assay. Cells were stained with DCFH-DA solution for 15 min and afterwards incubated with increasing concentrations of the extracts. 1 mM H_2O_2 was used as a positive control for verification of signal induction. Data presented

is the mean + SD of 4-5 independent measurements expressed as T/C of the fluorescence signal relative to the solvent control (Ctrl, 0.5% DMSO). Significant differences to the control were determined with one-sample Student's *t*-test (* p<0.05, ** p<0.01, *** p<0.001).

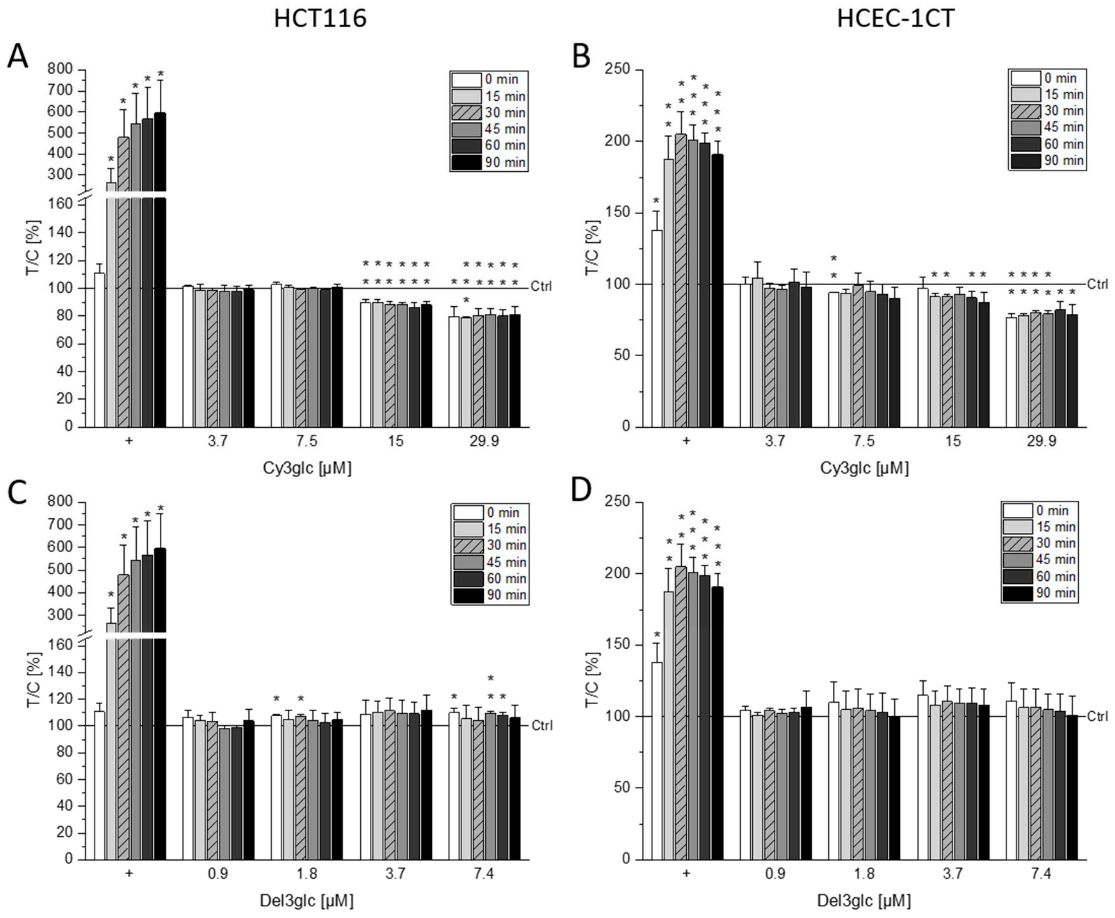

Figure S9: ROS-dependent induction of oxidative stress in HCT116 (left) and HCEC-1CT (right) cells after exposure to Cy3glc (**A, B**) and Del3glc (**C, D**) from 0-90 min measured with the DCF assay. Cells were stained with DCFH-DA solution for 15 min and afterwards incubated with increasing concentrations of anthocyanins. 1 mM H_2O_2 was used as a positive control for verification of signal induction. Data presented is the mean + SD of 4-5 independent measurements expressed as T/C of the fluorescence signal relative to the solvent control (Ctrl, 0.5% DMSO). Significant differences to the control were determined with one-sample Student's *t*-test (* p<0.05, ** p<0.01, *** p<0.001).

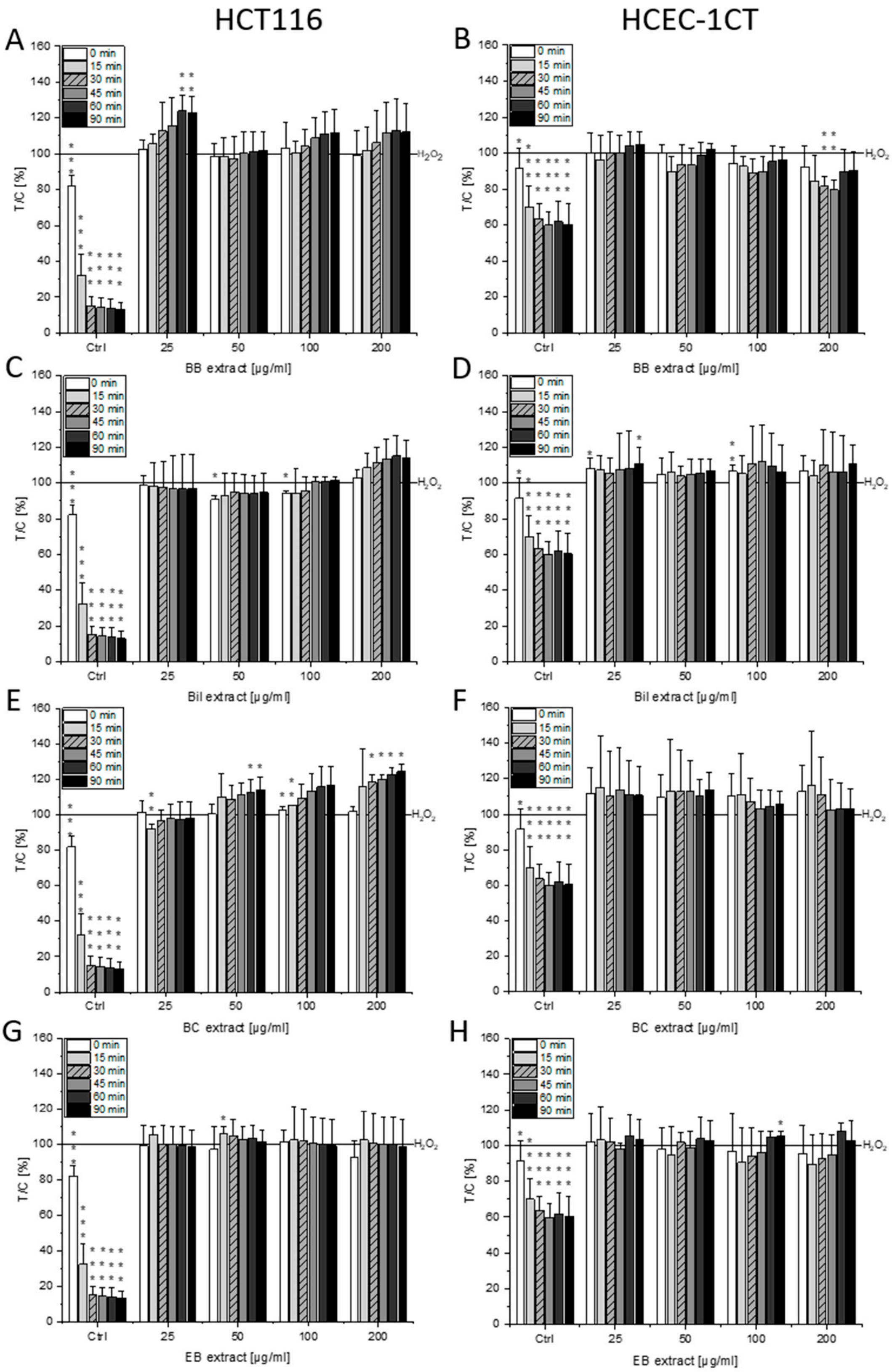

Figure S10: Effect of anthocyanin-rich extracts on H_2O_2-dependent ROS induction in HCT116 (left) and HCEC-1CT (right) cells from 0-90 min measured with the pDCF assay. Cells were pre-incubated for 24 h with extracts, stained with DCFH-DA solution for 15 min and subsequently challenged with 1 mM H_2O_2. Results presented are the means + SD of 4-6 biological replicates evaluated as T/C of the fluorescence

intensity signal relative to the H_2O_2 control after each time point. Cells treated only with 0.5% DMSO were used as control for proper signal induction by H_2O_2. Significant differences to the H_2O_2 control were calculated with one-sample Student's *t*-test (* p<0.05, ** p<0.01, *** p<0.001).

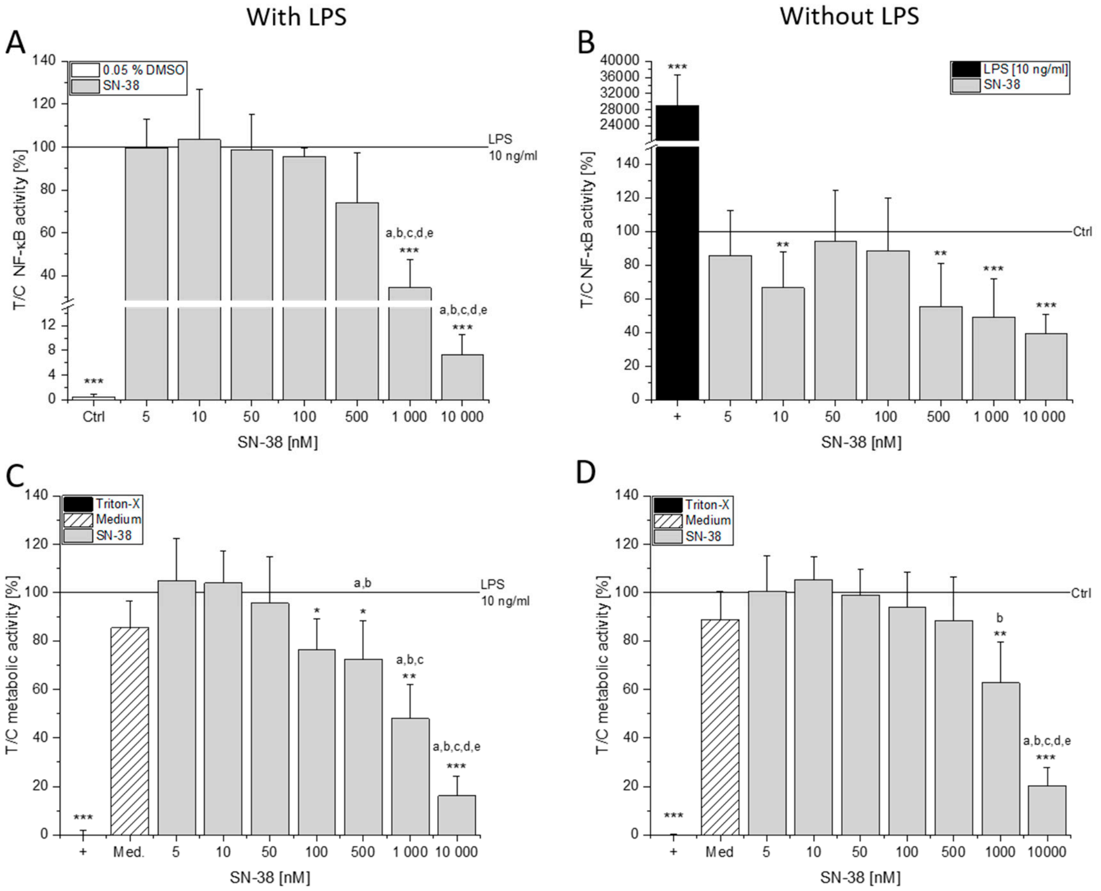

Figure S11: Immunomodulatory (**A, B**) and cytotoxic (**C, D**) effects of SN-38 with (left) and without (right) additional stimulation with 10 ng/ml LPS. THP1-Lucia™ cells were either pre-incubated with SN-38 for 2 h and subsequently additionally challenged with 10 ng/ml LPS for 18 h or incubated for 20 h with SN-38 alone. A solvent control or LPS control served as positive controls for functionality of the NF-κB assay. TX (0.1%) was used as positive control in the CTB assay. Results shown are the means + SD of 5-7 independent experiments expressed as T/C in % to the respective controls. Statistically significant differences to the control were calculated with one-sample Student's *t*-test (* p<0.05, ** p<0.01, *** p<0.001). Differences among the tested concentrations were determined with one-way ANOVA (p<0.05, a-e).

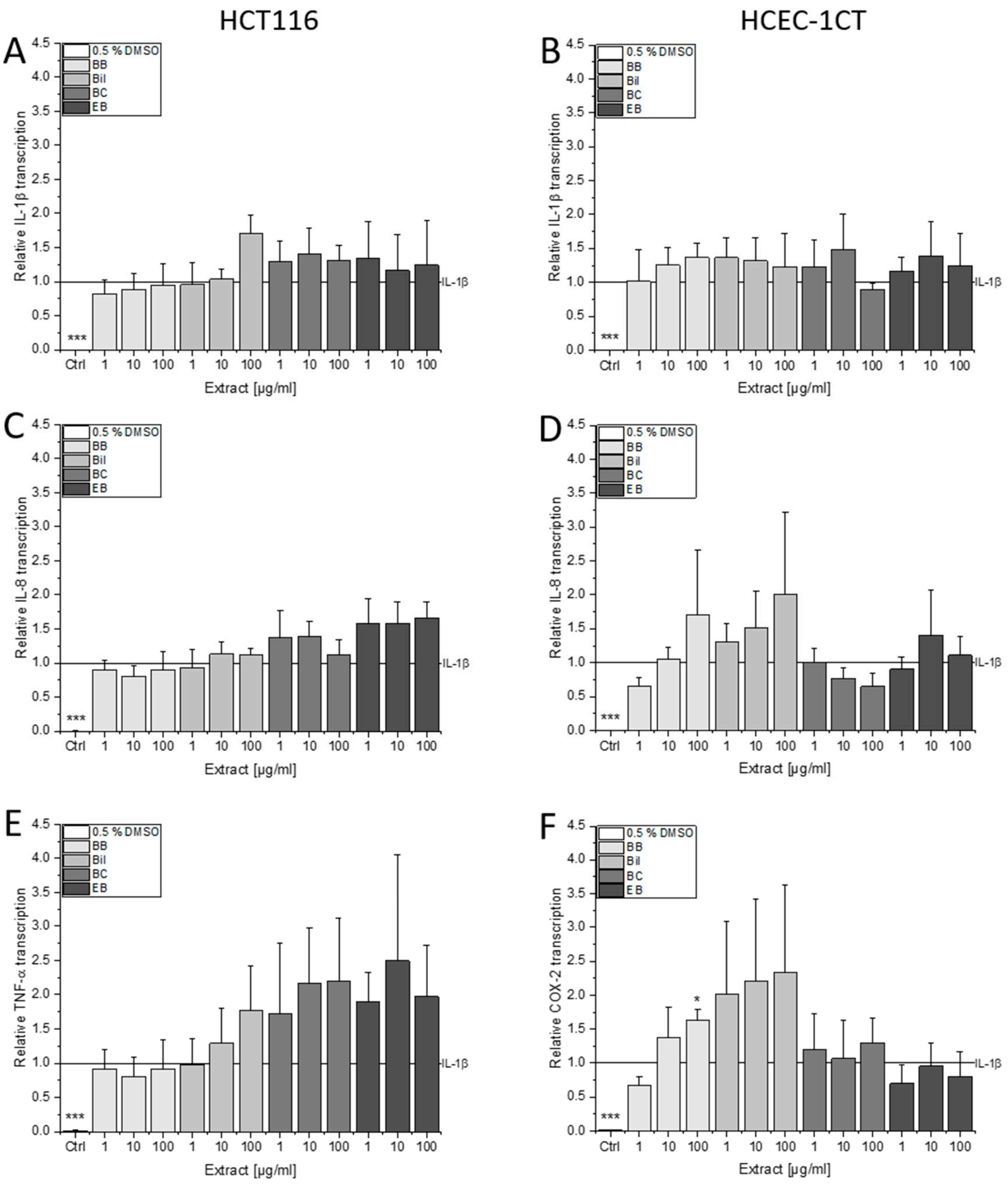

Figure S12: Relative gene transcription of IL-1β (**A, B**), IL-8 (**C, D**), TNF-α (**E**) or COX-2 (**F**) in HCT116 (left) and HCEC-1CT (right) cells measured with qRT-PCR. Cells were exposed to extracts for 2 h and subsequently additionally challenged with IL-1β protein (25 ng/ml) for 3 h. Data shown is the mean + SD of three independent measurements performed in technical duplicates. Results are the relative transcript levels evaluated with the $2^{-\Delta\Delta Ct}$ method normalized to the housekeeping genes (GAPDH and HPRT1) relative to the IL-1β control (calibrator, y=1). Differences to the IL-1β control were determined with two-sample Student's *t*-test (* $p<0.05$, *** $p<0.001$).
